# Phylogeographic Dynamics of Influenza A(H9N2) Virus Crossing Egypt

**DOI:** 10.3389/fmicb.2020.00392

**Published:** 2020-03-24

**Authors:** Ruiyun Li, Amany Adel, Jon Bohlin, Åke Lundkvist, Björn Olsen, John H.-O. Pettersson, Mahmoud M. Naguib

**Affiliations:** ^1^MRC Centre for Global Infectious Disease Analysis, Department of Infectious Disease Epidemiology, School of Public Health, Faculty of Medicine, Imperial College London, London, United Kingdom; ^2^National Laboratory for Veterinary Quality Control on Poultry Production, Animal Health Research Institute, Agriculture Research Center, Giza, Egypt; ^3^Department of Infectious Disease Epidemiology and Modelling, Domain for Infection Control and Environmental Health, Norwegian Institute of Public Health, Oslo, Norway; ^4^Department of Medical Biochemistry and Microbiology, Zoonosis Science Center, Uppsala University, Uppsala, Sweden; ^5^Department of Medical Sciences, Zoonosis Science Center, Uppsala University, Uppsala, Sweden; ^6^Marie Bashir Institute for Infectious Diseases and Biosecurity, Charles Perkins Centre, School of Life & Environmental Sciences and Sydney Medical School, The University of Sydney, Sydney, NSW, Australia

**Keywords:** avian influenza, H9N2, phylogeography, virus evolution, Egypt

## Abstract

Low pathogenic avian influenza (LPAI) virus of subtype H9N2 is the most frequently detected subtype among domestic poultry and is a public health concern because of its zoonotic potential. Due to the multiple and complex routes of LPAIV H9N2 between geographic regions, little is known about the spatial diffusion of H9N2 virus to, within, and from Egypt, where it is endemic among poultry since 2011. Using close to 800 publicly available hemagglutinin (HA) segment nucleotide sequences, associated location and temporal data, we conducted a Bayesian discrete phylogeographic analysis. Here, we reconstructed and traced the origin, spread and principal transmission routes of H9N2 across large geographical regions, in addition to the transmission between Egypt and the rest of the world and between different Egyptian governorates. Our analysis suggests that during the last few decades, H9N2 has been introduced back and forth continuously between the countries where it is endemic. Amongst these regions, Saudi Arabia, United Arab Emirates and Iraq act as main distribution hubs and drive the viral migration worldwide, with bi-directional and long-distance diffusions. It is noteworthy that H9N2 was introduced once to Egypt via Israel in mid 2009, and that the descendants of the Egyptian LAIVs H9N2 were back-transmitted to Israel in 2015. Additionally, governorates in middle Egypt (Giza, Fayoum and Bani Souwaif) are major hubs in the LPAIV H9N2 transmission network in Egypt. This knowledge highlights that H9N2 is both a global and a national concern and can aid in updating the surveillance program and vaccine strain selection.

## Introduction

Low pathogenic avian influenza virus (LPAIV) of subtype H9N2 is a subtype of influenza A viruses that circulate among different bird species and possess a public health concern because of its zoonotic potential (Peacock et al., 2019). Based on their genetic features, H9N2 viruses are classified into two major lineages, i.e., the “North American” and “Eurasian” lineages, circulating in poultry and wild birds. The Eurasian lineage is subdivided into three major lineages: 1-Korean lineage: A/duck/Hong Kong/Y439/97 (Y439-like) and A/chicken/Korea/38349-p96323/96 (Korean-like); 2-Y280 lineage: A/duck/Hong Kong/Y280/97-like (Y280-like), A/Chicken/Hong Kong/G9/97 (G9-like) and A/Chicken/Beijing/1/94 (BJ94-like); and 3-G1 lineage: A/quail/Hong Kong/G1/97-like (G1-like) (Guo et al., 2000). Since its emergence in 1996 and according to the clustering scheme described by Fusaro et al. (2011), the G1-like lineage displays the largest geographic spread extending from Eastern Asia to the Middle East. Further, the G1-like H9N2 viruses circulating in Central-Asian and Middle Eastern countries, have been diversified into four distinct groups (A, B, C, and D) (Fusaro et al., 2011; Naguib et al., 2017). Although H9N2 is endemic in many Asian and African countries, little is known about the ongoing viral diversifications and spatial expansions.

In particular, Egypt has experienced incursion of LPAI H9N2 viruses of the G1-like clade in late 2010 (Monne et al., 2012). Since then, LPAIV H9N2 has established endemic status among the Egyptian poultry population, causing serious economic problems to the poultry industry. In addition, the co-circulation with other endemic highly pathogenic AIVs (HPAIVs) of subtypes H5N1 and H5N8, has also been reported in wide parts of the Egyptian poultry population (Arafa et al., 2012; Kim, 2018; Samy and Naguib, 2018). In 2014, a reassortant LPAIV H9N2 was reported in pigeon containing five internal gene segments (PB2, PB1, PA, NP, and NS) form Eurasian AIV subtypes (Kandeil et al., 2017). The same genotype was found in backyard chickens in three Egyptian governorates in 2015 (Samir et al., 2019). Recently a new genotype was described similar to the reassortant H9N2 but inherited its NP gene segment from the Egyptian 2010 H9N2 viruses (Kandeil et al., 2019). It is also noteworthy that the continuous spread and circulation of LPAIV H9N2 in the Egyptian poultry population has been linked LPAIV H9N2 cases in humans and pigs (World Health Organization [WHO], 2016; Gomaa et al., 2018). In January 2015, three human cases of LPAI H9N2 following exposure to infected poultry was recorded indicating the zoonotic potential of these viruses (World Health Organization [WHO], 2016). Importantly, this ranks Egypt as the third country, after China and Bangladesh, reporting H9N2 infections in humans. The vast majority of the Egyptian H9N2 viruses encoded amino acid residues H183 and L226 (H3 numbering), the same feature as a vast majority of viruses in the G1-like lineage, which are described to correlate with a shift in affinity of the HA from the “avian” alpha 2–3 toward the “human-like” alpha 2–6 sialic acid linkage (Matrosovich et al., 2001; Naguib et al., 2017). LPAIVs H9N2 in Egypt form the majority of group B, G1 lineage along with viruses from Saudi Arabia, United Arab Emirates, Jordon, Israel, and Bangladesh (Kayali et al., 2014; Naguib et al., 2015). Phylogenetically, the Egyptian LPAIVs H9N2 have been described to be diversified further into three groups clustered within G1-B lineage based on their HA gene segment (Kandeil et al., 2014; Naguib et al., 2017). It is generally acknowledged that waterfowl migration and poultry trade play an important role in the spread of AIVs (Peacock et al., 2019). However, the geographic diffusion of H9N2 globally and into and within Egypt is not fully understood. In particular, the probability of backward transmission of Egyptian H9N2 viruses to the rest of the world is still unclear.

Understanding virus spread and migration patterns could aid in better preparation for epidemics and provide useful guidelines for virus control. In this study, we conducted a phylogeographic analysis of LPAIVs H9N2 obtained from our institutional repositories and public databases, including >700 viruses from 1997–2018, to illustrate the dissemination of LPAIVs H9N2 from far Asia to the Middle East and North Africa, in addition to uncovering the transmission pathways of LPAIVs H9N2 in Egypt. The findings of this study should contribute to the strategic surveillance and targeted planning of control and prevention activities of not only LPAIVs H9N2 but also other subtypes co-circulating in Egypt.

## Materials and Methods

### Sequence Data Collection and Phylogenetic Analysis

Initially, the genetic sequence data of more than 3,500 HA segments of global H9N2 viruses in 1997–2018 were retrieved from GenBank and Global Initiative on Sharing All Influenza Data (GISAID) platforms. To reduce the dataset size, several steps were taken. Initially, identical sequences were removed by keeping the sequence with the earliest date. Secondly, a maximum-likelihood tree was computed using PhyML v.3.0 (Guindon et al., 2009), employing the GTR + g model of nucleotide substitution, whereafter sequences from high-density monophyletic clades from China were subsampled to reflect the current diversity. With this reduced dataset, new maximum-likelihood tree, using the same approach as above, was computed. Temporal structure was subsequently assessed with TempEst v.1.5.1 (Rambaut et al., 2016) after which additional sequences were excluded. The final dataset contained 740 representative sequences (alignment length of 1,738 nucleotides) with the corresponding virus name, host, collection time and geographical location. The majority of samples were distributed in Bangladesh, Israel and Egypt, with over 120 sequences in each country. By contrast, less than 10 samples were obtained in Iraq, Lebanon, Libya, Morocco, and Tunisia. More details on sample distribution in Supplementary Table S1. Details on sequences in the final dataset re-provided in Supplementary Table S6 where those previously generated in our institutional repositories are marked bold.

### Global H9N2 Phylogeographic Pathways

To investigate the main global-level spatial diffusion routes between 1997 and 2018, locations were assigned to different regions: Middle East 1 (Saudi Arabia, United Arab Emirates and Iraq), Middle East 2 (Jordan, Lebanon and Israel), North Africa (Egypt, Morocco, Libya, Tunisia, and Algeria), China, Iran and South Asia (Bangladesh, India and Pakistan) (Supplementary Table S1). In a parallel analysis, Egypt was designated as a separate region from other North African countries (Morocco, Libya, Tunisia, and Algeria) to prioritize viral migrations between Egypt and the rest regions. The reconstruction of viral migration network was conducted through a Bayesian statistical inference framework using a Bayesian Markov chain Monte Carlo (MCMC) method available in the BEAST package (version 1.7.5) (Drummond et al., 2012). More specifically, a discrete phylogeography was inferred using the general time reversible (GTR) substitution model and a constant size coalescent prior. Three MCMC chains were run independently for 100,000,000 iterations and sampled every 10,000 steps. The convergence of MCMC was assessed by the effective sample size (ESS) of at least 200 using Tracer. Following a burn-in of 10% of each run, the three chains were combined and resampled to 1,000 trees to create the maximum clade credibility (MCC) tree. The reconstructed MCC tree was visualized and edited using FigTree (version 1.4.0). ‘Markov jump’ counts was used to quantify the expected number of transitions among different locations (Minin and Suchard, 2008). Additionally, the Bayesian stochastic search variable selection (BSSVS) was applied to undertake the Bayes factor (BF) test and estimate the transition rates. We assumed Poisson prior for the BSSVS, assigning 50% prior probability to the minimal rate configuration (Lemey et al., 2009). The strength of transition rates was assessed using SPREAD (version 1.0.6) (Bielejec et al., 2011) with a Bayes factor threshold of ≥ 3. The region with the highest bayes factor support was denoted as the most probable transmission hub for global viral migrations. To investigate the time when the inter-regional transmission between Israel and Egypt occurred, the time to the most recent common ancestors and 95% HPD intervals of the collected Israeli and Egyptian H9N2 viruses were also estimated. Given the spatial-temporal heterogeneity in viral isolation, temporal variation of transmission rate was not evaluated.

### Egyptian H9N2 Phylogeographic Pathways

In total 97 out of 171 available Egyptian sequences had known governorate information and were used to reconstruct the diffusing pattern within Egypt (Supplementary Table S2). By using the same molecular clock model, nucleotide substitution model and coalescent prior as above, two MCMC chains were run independently for 100,000,000 iterations and sampled every 5,000 steps. Governorates with the highest bayes factor support is denoted as the most probable transmission hub of viral migration within Egypt. To facilitate the interpretation of the results, governorates were categorized into three areas, i.e., Lower Egypt (the Nile Delta), Middle Egypt (including Giza, Fayoum and Bani Souwaif) and Upper Egypt.

## Results

### Global Hierarchical Viral Diffusion Network

**FIGURE 1:**
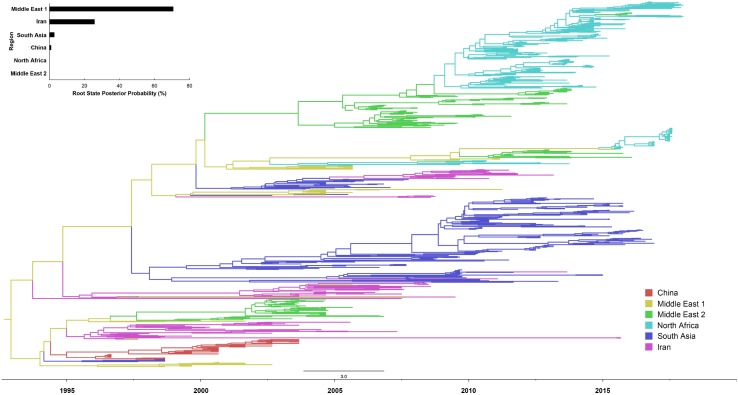
Bayesian evolutionary tree of global LPAI H9N2 viruses based on the nucleotide sequence of the HA gene between 1997 and 2018. Locations were assigned to six regions: Middle East 1 (Saudi Arabia, United Arab Emirates and Iraq), Middle East 2 (Jordan, Lebanon and Israel), Africa (Egypt, Morocco, Libya, Tunisia and Algeria), East Asia (China), West Asia (Iran), and South Asia (Bangladesh, India and Pakistan). The posterior probability of each location acting as transmission hub, i.e., root state posterior probability, was also presented.

**FIGURE 2:**
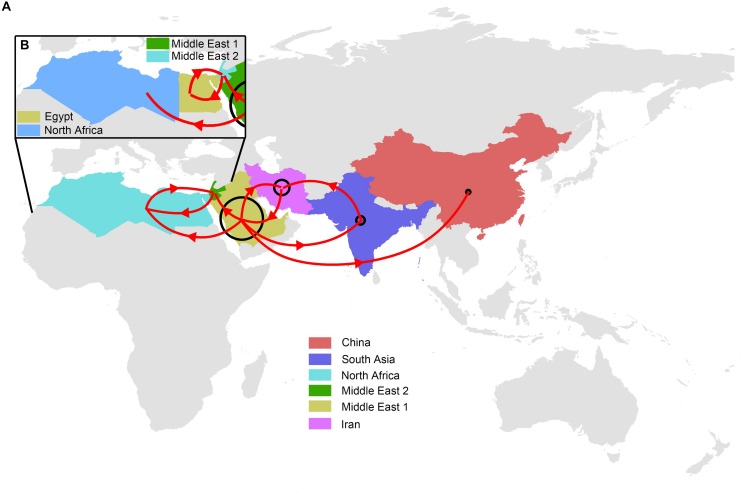
Migration pathways of global LPAI H9N2 viruses. **(A)** Locations were assigned to six regions: Middle East 1 (Saudi Arabia, Emirates and Iraq), Middle East 2 (Jordan, Lebanon and Israel), North Africa (Egypt, Morocco, Libya, Tunisia and Algeria), China, Iran and South Asia (Bangladesh, India and Pakistan). **(B)** Egypt was designated as a separate region from North African countries (Morocco, Libya, Tunisia and Algeria). Lines with arrows illustrate main migration routes. The size of the circle indicates relative chance of a region acting as a migration source. The color of each region is consistent with those shown in Figure 1.

Our reconstruction of the H9N2 transmission pathways shows that the propagation network of H9N2 is highly structured, with significant inter-regional expansions with distinct source-sink dynamics (Figures 1, 2 and Supplementary Table S3). Amongst these regions investigated, Middle East 1 was shown to be a major inter-regional transmission hub of H9N2 viruses, as indicated by the much higher posterior probability (>70%), linking viruses isolated from all other regions (except for Egypt). Similarly, Iran was also shown to be important in the global spread and maintenance of H9N2, although not in the same magnitude as Middle East 1. By contrast, the lack of posterior probability support for Middle East 2, Egypt and North Africa suggests that they are less likely to act as sources of H9N2 in other regions. Furthermore, bi-directional transmissions were inferred between some regions, i.e., North Africa and Middle East 2, and Middle East 1 and Iran, which may due primarily to their spatial adjacency. In particular, Egyptian viruses were closely related to those isolated from Jordan, Lebanon and Israel, with bi-directional viral migrations between these two regions (Figure 2 and Supplementary Table S4). However, when viewed in detail [Supplementary Figure S1 and given the sample size (Supplementary Table S1)], the Egyptian H9N2 was most likely introduced via Israel around mid 2009 (95% HPD: March 2009–October 2009). Subsequently, H9N2 was back-transmitted from Egypt to Israel ca. 2015 (95% HPD: December 12, 2014–December 30, 2015).

### Transmission Pathways of H9N2 in Egypt

**FIGURE 3:**
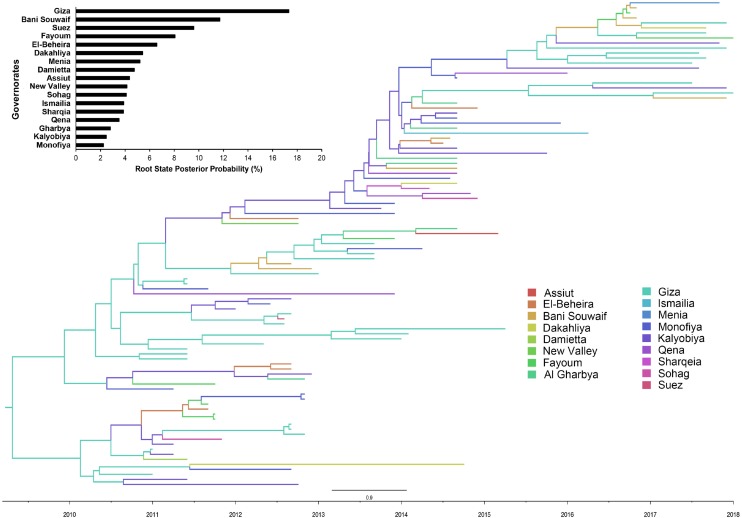
Bayesian evolutionary tree of Egyptian LPAI H9N2 viruses based on the nucleotide sequence of the HA gene between 2010 to 2018. The posterior probability of each governorate as transmission hub, i.e., root state posterior probability, were also presented.

**FIGURE 4:**
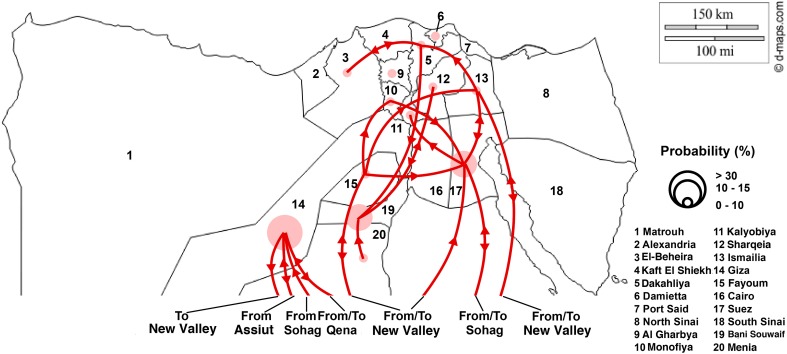
Migration pathways of LPAI H9N2 viruses in Egypt. Lines with arrows illustrate main migration routes between the different governorates (1–20). The size of the circle indicates relative chance of a governorate acting as a migration source.

There is a distinct spatial viral diffusion structure within Egypt, including both intra- and inter-area transmission pathways (Figures 3, 4 and Supplementary Table S5). Intra-area viral diffusions were primarily concentrated in Lower Egypt, with bi-directional transmission between El-Beheira and Dakahliya. Additionally, inter-area transmissions are generally migrated from Middle or Upper Egypt to Lower Egypt. It is noteworthy that governorates located in the Middle Egypt, i.e., Giza, Fayoum and Bani Souwaif, are most likely transmission hubs, linking viral migrations between Lower and Upper Egypt. In the interim, direct long-distance diffusions between Upper and Lower Egypt are also observed, particularly the Suez – Sohag and Ismailia – New Valley viral migrations. Of note, transmission from New Valley to Fayoum is of the highest significance compared with the others. Given the localized circulation and viral incursion from Upper and Middle Egypt, we suggest that Lower Egypt may be the reservoir of H9N2 viruses.

## Discussion

In Egypt, LPAIV H9N2 continues to cause a significant impact on poultry production industry since its introduction in 2010 (Kandeil et al., 2014; Naguib et al., 2015); in addition, it poses threat to the public health due to its zoonotic potential where subsequent human infections have been reported (Naguib et al., 2017; Nagy et al., 2017). Despite many reports on the molecular features, the transmission dynamics of LPAIVs H9N2 in Egypt is yet to be defined. In this study, we aimed, by using discrete state Bayesian phylogeography, to reveal the evolutionary history and spatial diffusion process of LPAIV H9N2 to, in, and from Egypt, which will in turn aid in establishing more effective avian influenza control strategies.

We find a hierarchical viral propagation network of LPAIV H9N2 being active since the past twenty years. Overall, there is a strong evidence of geographically structured viral diffusions both at the global and the regional scale. Saudi Arabia, United Arab Emirates and Iraq are likely to have played a dominant role in driving the global diffusions of LPAIV H9N2, which was supported by its highest probability as being the transmission sources and multiple transmission events to the rest of the world. This predominant role may potentially be due to its specific location, i.e., in the intersection of the Central Asian and Black Sea-Mediterranean flyways (Birdlife, 2019). It might be also possible that the increased probability of transmission reflects the commercial trade of poultry between regions.

The LPAIVs H9N2 isolated in North Africa has been demonstrated to share a close genetic relationship with those sampled from the Middle East (Peacock et al., 2019). Notably, we proposed a diverse genetic relationship between Africa and the Middle East, with distinct sources of LPAIVs H9N2 between Egypt and the rest of the North African countries. More specific, the close genetic relationship between viruses circulating in Egypt and the neighboring Middle Eastern countries indicated that Jordan, Lebanon and, in particular, Israel are the primary sources of Egyptian H9N2 viruses. By contrast, Saudi Arabia, Emirates and Iraq are most likely to be the source of H9N2 viruses in North Africa (Libya, Algeria, Tunisia, and Morocco). Given the different vaccination strategies implemented in African and Middle Eastern countries (Nagy et al., 2017), we suggest that these strategies may have facilitated the continuous evolution and diversification of LPAIVs H9N2, forming complicated genetic relationship among countries. Additionally, the lack of the direct wild bird movements (Birdlife, 2019) between Egypt and North Africa may have favored viral divergence between two regions as well.

Considering our analysis span more than 20 years of global H9N2 evolution, i.e., 1997–2018, the inferred transmission pathways and sources for the explored regions and time periods should more so be seen as a general pattern for the time-period as a whole. However, although we have subsampled all of the available H9N2 HA genetic diversity, it is important to note that reporting LPAIVs H9N2 varies between different countries our analysis is just reflecting the currently available data. Previous studies have shown extensive expansions to the Middle East after its first emergence in Asia, forming a close genetic linkage between two regions (Fusaro et al., 2011; Nagy et al., 2017; Wei and Li, 2018). Furthermore, phylogenetic divergence of Asian and the Middle Eastern H9N2 viruses has also been reported through the ongoing viral evolution (Fusaro et al., 2011; Hu et al., 2017; Naguib et al., 2017; Wei and Li, 2018). Together, these finding indicated that Asia served as the original transmission source of global H9N2 viruses, but our analyses give heighten the distinct role of Middle East as transmission hub in shaping the following spatial expansions.

From this study, it is observed that Egyptian LPAIVs H9N2 have evolved into three major groups (Supplementary Figure S2) and was shown to have been introduced from Israel in mid 2009 (Supplementary Figure S1), which is in accordance with a previous study (Naguib et al., 2015). Following this incursion, no further introduction of LPAIV H9N2 has been observed from other countries. Determinants of viral diffusion in Egypt differed markedly between locations, where rural population density (Delta regions) was found to be a strong disseminator for LPAIV H9N2 viral migration in Egypt. This shows that movement dynamics within the Nile Delta region of Egypt are complex and important for the understanding of H9N2 circulation in Egypt. The data in this study suggests that governorates in middle Egypt (Giza, Fayoum and Bani Souwaif) are the major hubs in the LPAIV H9N2 transmission network in Egypt and has a strong connection between upper and lower Egyptian governorates (Figure 4). The spread of LPAIV H9N2 across Egypt primarily occurs through movements of domestic poultry. Moreover, the phylogeographic networks of LPAIV H9N2 in Egypt, shown in this study, are likely to be indicative of poultry movements across Egypt. Therefore, our findings propose that transportation networks of live poultry between lower (Nile Delta) and upper regions should be considered key routes for LPAIV H9N2 dissemination in Egypt.

Taken together, our study showed the transmission of H9N2 influenza viruses to, in and from Egypt, is part of the complex migration network from far Asia to the Middle East to Africa. Data generated on LPAIVs H9N2 on the transmission patterns in our study can be informative for AIV control strategies for the local administrative authorities. Note that This inferred transmission pattern of HA gene should not be projected to other gene segments, given the different evolutionary and transmission dynamics. Continuous monitoring and characterization of AIV in Egypt is recommended to fully understand the transmission patterns of this virus.

## Data Availability Statement

The datasets generated for this study can be found in the GenBank (National Center for Biotechnology Information), Global Initiative on Sharing All Influenza Data (GISAID) platforms.

## Author Contributions

RL, JP, and MN conceived the study, produced, analyzed, and interpreted the phylogeographic data. AA, JB, and JP provided the epidemiological data and performed the viruses sub-sampling. RL and MN drafted the manuscript. All authors critically analyzed and revised the manuscript, and provided final approval.

## Conflict of Interest

The authors declare that the research was conducted in the absence of any commercial or financial relationships that could be construed as a potential conflict of interest.
