# Supplementary material for: Phylogeographic Dynamics of Influenza A(H9N2) Virus Crossing Egypt
Source: Front Microbiol. 2020 Mar 24;11:392. doi: 10.3389/fmicb.2020.00392 (PMC7105594; doi:10.3389/fmicb.2020.00392)
Supplement: Supplementary file 1 [file Data_Sheet_1.docx]

**Supplementary Figures**

**Figure S1. Estimates of the time to the most common ancestor for the Israeli and Egyptian H9N2 viruses.**Egypt was designated as a separated region from North Africa (Morocco, Libya, Tunisia and Algeria). All the rest regions remain same as those in Figure 1. The time to most recent common ancestor and 95%HPD of Israel and Egyptian viruses were shown by red dots and grey bars. The posterior probability of each location acting as transmission hub, i.e. root state posterior probability, was also presented.





**Figure S2. Phylogeny of HA segment of Egyptian LPAI H9N2 viruses between 2010 to 2018.** Phylogeny of the HA gene was inferred using the maximum likelihood method. All 171 Egyptian H9N2 viruses and the Hong Kong/G1/97 virus were included. Three groups (B1−B3) of G1-like viruses were distinguished by colour and highlighted by vertical lines. Bootstrap values >80 were shown on branches.

**

**

**Supplementary Tables**

**Table S1. Designation of global H9N2 viruses to different regions.** Sample size and countries in each region were shown. Sample size of Egypt, Jordan, Lebanon and Israel was also presented.

| region | Sample size | Countries |
| --- | --- | --- |
| East Asia | 41 | China (including Hong Kong Special Administrative Region) |
| Middle East1 | 40 | Saudi Arabia, United Arab Emirates, Iraq |
| Middle East2 | 147 | Jordan (12), Lebanon (3) and Israel (132) |
| Africa | 224 | Egypt (171), Morocco, Libya, Tunisia, Algeria |
| South Asia | 198 | Bangladesh, India, Pakistan |
| West Asia | 90 | Iran |
| Total | 740 |  |

**Table S2. Sample size of Egyptian H9N2 viruses.** 97 out of 171 samples with known governorate was assigned to a specific governorate.

| **Governorate** | **Sample size** |
| --- | --- |
| Assiut | 1 |
| El-Beheira | 6 |
| bani Souwaif | 7 |
| Dakahliya | 3 |
| Damietta | 1 |
| New Valley | 1 |
| Fayoum | 7 |
| Al Gharbya | 6 |
| Giza | 28 |
| Ismailia | 1 |
| Menia | 1 |
| Monofiya | 13 |
| Kalyobiya | 14 |
| Qena | 4 |
| Sharqeia | 1 |
| Sohag | 2 |
| Suez | 1 |

**Table S3. Details of global transmission pathways of H9N2 viruses.** Locations were assigned to six regions: Middle East 1 (Saudi Arabia, Emirates and Iraq), Middle East 2 (Jordan, Lebanon and Israel), North Africa (Egypt, Morocco, Libya, Tunisia and Algeria), China, Iran and South Asia (Bangladesh, India and Pakistan). BF value was shown to represent the significance of each pathway.

| ID | Pathway | | | BF value |
| --- | --- | --- | --- | --- |
| 1 | Middle East1 | 🡺 | South Asia | 158 |
| 2 | Middle East1 | 🡺 | Iran | 142 |
| 3 | Iran | 🡺 | Middle East1 | 88 |
| 4 | Middle East1 | 🡺 | Middle East2 | 57 |
| 5 | South Asia | 🡺 | Iran | 38 |
| 6 | Middle East1 | 🡺 | North Africa | 30 |
| 7 | Egypt | 🡺 | Middle East2 | 28 |
| 8 | Middle East2 | 🡺 | Egypt | 13 |
| 9 | Middle East1 | 🡺 | China | 6 |

**Table S4. Details of global transmission pathways of H9N2 viruses using alternative classification of regions.** Egypt is designated as a separate regional from North Africa (Morocco, Libya, Tunisia and Algeria). Designation of other regions remain the same as shown in Table S3.

| ID | Pathway | | | BF value |
| --- | --- | --- | --- | --- |
| 1 | Middle East1 | 🡺 | South Asia | 1098 |
| 2 | Middle East1 | 🡺 | Iran | 637 |
| 3 | Iran | 🡺 | Middle East1 | 428 |
| 4 | Middle East1 | 🡺 | Middle East2 | 338 |
| 5 | South Asia | 🡺 | Iran | 315 |
| 6 | Middle East1 | 🡺 | North Africa | 138 |
| 7 | Egypt | 🡺 | Middle East2 | 61 |
| 8 | Middle East2 | 🡺 | Egypt | 21 |
| 9 | Middle East1 | 🡺 | China | 7 |

**Table S5. Details of transmission pathways of H9N2 in Egypt.** Transmissions were classified as different types based on their spatial areas, i.e. intra- and inter-area transmissions.

| Transmission type | Area | ID | Pathway | BF |
| --- | --- | --- | --- | --- |
| Intra-area | Lower | 1 | Monofiya 🡺 Suez | 83.1 |
|  | Upper | 2 | Assiut 🡺 New Valley | 45.29 |
|  | Lower | 3 | Dakahliya 🡺 Beheira | 41.3 |
|  | Lower | 4 | Beheira 🡺 Dakahliya | 40.68 |
|  | Upper | 5 | Assiut 🡺 Qena | 40.47 |
|  | Lower | 6 | Ismailia 🡺 Suez | 27.56 |
|  | Lower | 7 | Ismailia 🡺 Dakahliya | 23.48 |
|  | Lower | 8 | Suez 🡺 Kalyobiya | 3.01 |
| Inter-area | Upper - Middle | 1 | New Valley 🡺 Fayoum | 230.94 |
|  | Middle - Upper | 2 | Giza 🡺 Assiut | 145.67 |
|  | Upper - Middle | 3 | Assiut 🡺 Giza | 122.19 |
|  | Middle - Upper | 4 | Fayoum 🡺 New Valley | 89.41 |
|  | Middle - Lower | 5 | Bani Souwaif 🡺 Sharqeia | 89.25 |
|  | Lower - Upper | 7 | Suez 🡺 Sohag | 76 |
|  | Middle - Upper | 8 | Giza 🡺 New Valley | 71.81 |
|  | Upper - Middle | 9 | Sohag 🡺 Giza | 61.73 |
|  | Middle - Lower | 10 | Fayoum 🡺 Ismailia | 55.45 |
|  | Upper - Lower | 11 | Ismailia 🡺 New Valley | 50.47 |
|  | Upper - Lower | 12 | Suez 🡺 Ismailia | 38.34 |
|  | Upper - Lower | 13 | New Valley 🡺 Ismailia | 34.42 |
|  | Lower - Middle | 14 | Dakahliya 🡺 Bani Souwaif | 30.56 |
|  | Upper - Middle | 15 | Minya 🡺 Bani Souwaif | 29.35 |
|  | Middle - Lower | 16 | Fayoum 🡺 Monofiya | 20.56 |
|  | Upper - Lower | 17 | Sohag 🡺 Suez | 13.29 |
|  | Middle - Upper | 18 | Giza 🡺 Qena | 13.25 |
|  | Upper - Lower | 19 | New Valley 🡺 Suez | 12.3 |
|  | Upper - Middle | 20 | Qena 🡺 Giza | 10.39 |
|  | Lower - Middle | 21 | Sharqeia 🡺 Bani Souwaif | 9.34 |
|  | Middle - Lower | 22 | Fayoum 🡺 Suez | 3 |

**Table S6. Details of sequences analysed in the study.** The details, including accession number, virus name, and the country and time of isolation, of H9N2 sequences analysed in the study are provided.

| Accession | Country | Virus name | Time of isolation |
| --- | --- | --- | --- |
| MK788283 | Algeria | A/chicken/Algeria/10BBD/2017 | 2017-04 |
| MK788282 | Algeria | A/chicken/Algeria/12BBD/2017 | 2017-04 |
| MK788281 | Algeria | A/chicken/Algeria/13BBD/2017 | 2017-04 |
| MK788280 | Algeria | A/chicken/Algeria/15BBD/2017 | 2017-04 |
| MK788279 | Algeria | A/chicken/Algeria/17BBD/2017 | 2017-04 |
| MK788278 | Algeria | A/chicken/Algeria/18BBD/2017 | 2017-04 |
| MK788277 | Algeria | A/chicken/Algeria/19BBD/2017 | 2017-04 |
| MK788276 | Algeria | A/chicken/Algeria/201/2017 | 2017-04 |
| MK788275 | Algeria | A/chicken/Algeria/203/2017 | 2017-04 |
| MK788274 | Algeria | A/chicken/Algeria/204/2017 | 2017-04 |
| MK240300 | Algeria | A/chicken/Algeria/205/2017 | 2017-12-05 |
| MK240296 | Algeria | A/chicken/Algeria/206/2017 | 2017-12-05 |
| MK240295 | Algeria | A/chicken/Algeria/207/2017 | 2017-12-05 |
| MK240291 | Algeria | A/chicken/Algeria/208/2017 | 2017-12-05 |
| MK240289 | Algeria | A/chicken/Algeria/209/2017 | 2017-12-05 |
| MK240282 | Algeria | A/chicken/Algeria/210/2017 | 2017-12-05 |
| MK240278 | Algeria | A/chicken/Algeria/211/2017 | 2017-12-05 |
| MK240271 | Algeria | A/chicken/Algeria/212/2017 | 2017-12-05 |
| MK240270 | Algeria | A/chicken/Algeria/213/2017 | 2017-12-05 |
| MK240268 | Algeria | A/chicken/Algeria/214/2017 | 2017-12-05 |
| MK240266 | Algeria | A/chicken/Algeria/215/2017 | 2017-11-14 |
| MK240264 | Algeria | A/chicken/Algeria/216/2017 | 2017-12-05 |
| MK240261 | Algeria | A/chicken/Algeria/219/2017 | 2017-12-05 |
| MK240260 | Algeria | A/chicken/Algeria/220/2017 | 2017-11-13 |
| MK240259 | Algeria | A/chicken/Algeria/221/2017 | 2017-12-05 |
| MK240257 | Algeria | A/chicken/Algeria/222/2017 | 2017-11-13 |
| MK240256 | Algeria | A/chicken/Algeria/223/2017 | 2017-11-14 |
| MK240252 | Algeria | A/chicken/Algeria/224/2017 | 2017-11-13 |
| MK240247 | Algeria | A/chicken/Algeria/225/2017 | 2017-11-14 |
| MK240244 | Algeria | A/chicken/Algeria/226/2017 | 2017-11-13 |
| MK240240 | Algeria | A/chicken/Algeria/227/2017 | 2017-11-14 |
| MK240233 | Algeria | A/chicken/Algeria/228/2017 | 2017-11-14 |
| MK240230 | Algeria | A/chicken/Algeria/229/2017 | 2017-11-13 |
| MK240228 | Algeria | A/chicken/Algeria/230/2017 | 2017-11-13 |
| MK240226 | Algeria | A/chicken/Algeria/6BBD/2017 | 2017-11-13 |
| MK240224 | Algeria | A/chicken/Algeria/8BBD/2017 | 2017-11-14 |
| MK240223 | Algeria | A/chicken/Algeria/9BBD/2017 | 2017-11-13 |
| KC986294 | Bangladesh | A/chicken/Bangladesh/VP01/2006 | 2006-09 |
| JX273539 | Bangladesh | A/chicken/Bangladesh/627/2007 | 2007 |
| JX273540 | Bangladesh | A/chicken/Bangladesh/FDIL_M_112/2007 | 2007 |
| KC757967 | Bangladesh | A/chicken/Bangladesh/2075/2009 | 2009-03-22 |
| KC757788 | Bangladesh | A/duck/Bangladesh/1231/2009 | 2009-02-03 |
| KC757885 | Bangladesh | A/environment/Bangladesh/907/2009 | 2009-01-07 |
| KF188249 | Bangladesh | A/quail/Bangladesh/907/2009 | 2009 |
| KC757999 | Bangladesh | A/chicken/Bangladesh/5209/2009 | 2009-12-27 |
| KC757975 | Bangladesh | A/pigeon/Bangladesh/4303/2009 | 2009-08-10 |
| KC757983 | Bangladesh | A/environment/Bangladesh/5144/2009 | 2009-12-27 |
| KC757832 | Bangladesh | A/chicken/Bangladesh/8411/2010 | 2010-08-06 |
| KC758030 | Bangladesh | A/chicken/Bangladesh/8725/2010 | 2010-09-01 |
| KC757855 | Bangladesh | A/chicken/Bangladesh/9334/2010 | 2010-11-15 |
| KC757848 | Bangladesh | A/environment/Bangladesh/9350/2010 | 2010-11-15 |
| KC758067 | Bangladesh | A/environment/Bangladesh/9306/2010 | 2010-11-15 |
| KC757991 | Bangladesh | A/chicken/Bangladesh/8996/2010 | 2010-10-15 |
| KC757951 | Bangladesh | A/chicken/Bangladesh/9029/2010 | 2010-10-15 |
| KC757907 | Bangladesh | A/environment/Bangladesh/9457/2010 | 2010-12-09 |
| KC757863 | Bangladesh | A/environment/Bangladesh/5745/2010 | 2010-01-20 |
| KC758060 | Bangladesh | A/chicken/Bangladesh/8413/2010 | 2010-08-06 |
| KC757937 | Bangladesh | A/environment/Bangladesh/8463/2010 | 2010-09-01 |
| KC758023 | Bangladesh | A/environment/Bangladesh/8465/2010 | 2010-09-01 |
| KC758007 | Bangladesh | A/environment/Bangladesh/8202/2010 | 2010-08-07 |
| KC757915 | Bangladesh | A/chicken/Bangladesh/10401/2011 | 2011-02-19 |
| KC757840 | Bangladesh | A/chicken/Bangladesh/10450/2011 | 2011-02-19 |
| KC758106 | Bangladesh | A/chicken/Bangladesh/11309/2011 | 2011-06-30 |
| KC757817 | Bangladesh | A/environment/Bangladesh/12077/2011 | 2011-08-14 |
| KC758015 | Bangladesh | A/chicken/Bangladesh/10897/2011 | 2011-05-29 |
| KC757893 | Bangladesh | A/chicken/Bangladesh/11154/2011 | 2011-06-28 |
| KC758083 | Bangladesh | A/environment/Bangladesh/10234/2011 | 2011-02-08 |
| KC758091 | Bangladesh | A/environment/Bangladesh/10307/2011 | 2011-02-19 |
| KC757959 | Bangladesh | A/environment/Bangladesh/10306/2011 | 2011-02-19 |
| KC758114 | Bangladesh | A/environment/Bangladesh/10313/2011 | 2011-02-19 |
| KC757922 | Bangladesh | A/environment/Bangladesh/10316/2011 | 2011-02-19 |
| KC758075 | Bangladesh | A/environment/Bangladesh/11597/2011 | 2011-07-20 |
| KC757870 | Bangladesh | A/environment/Bangladesh/12103/2011 | 2011-08-14 |
| KC757943 | Bangladesh | A/environment/Bangladesh/12116/2011 | 2011-08-14 |
| KC757929 | Bangladesh | A/environment/Bangladesh/12119/2011 | 2011-08-14 |
| KC757796 | Bangladesh | A/environment/Bangladesh/12068/2011 | 2011-08-14 |
| KC758099 | Bangladesh | A/environment/Bangladesh/11173/2011 | 2011-06-28 |
| KC758037 | Bangladesh | A/chicken/Bangladesh/13962/2011 | 2011-11-24 |
| KC757824 | Bangladesh | A/chicken/Bangladesh/13916/2011 | 2011-11-24 |
| KJ643800 | Bangladesh | A/environment/Bangladesh/18530/2012 | 2012-11-25 |
| KJ643643 | Bangladesh | A/quail/Bangladesh/18521/2012 | 2012-11-25 |
| KJ643792 | Bangladesh | A/quail/Bangladesh/18514/2012 | 2012-11-25 |
| KJ643651 | Bangladesh | A/quail/Bangladesh/18210/2012 | 2012-10-21 |
| KJ643848 | Bangladesh | A/chicken/Bangladesh/18224/2012 | 2012-10-21 |
| KJ643784 | Bangladesh | A/chicken/Bangladesh/18408/2012 | 2012-11-15 |
| KJ643832 | Bangladesh | A/environment/Bangladesh/18311/2012 | 2012-10-21 |
| KJ643636 | Bangladesh | A/environment/Bangladesh/18315/2012 | 2012-10-21 |
| KJ643708 | Bangladesh | A/environment/Bangladesh/18317/2012 | 2012-10-21 |
| KJ643620 | Bangladesh | A/chicken/Bangladesh/18549/2012 | 2012-11-20 |
| KJ643824 | Bangladesh | A/chicken/Bangladesh/16448/2012 | 2012-03-22 |
| KJ643721 | Bangladesh | A/chicken/Bangladesh/18460/2012 | 2012-11-15 |
| KJ643864 | Bangladesh | A/environment/Bangladesh/18846/2013 | 2013-01-28 |
| KJ643628 | Bangladesh | A/environment/Bangladesh/18849/2013 | 2013-01-28 |
| KJ643763 | Bangladesh | A/environment/Bangladesh/19842/2013 | 2013-05-25 |
| KJ643816 | Bangladesh | A/environment/Bangladesh/20199/2013 | 2013-07-08 |
| KJ643692 | Bangladesh | A/quail/Bangladesh/20177/2013 | 2013-07-08 |
| KJ643747 | Bangladesh | A/quail/Bangladesh/19475/2013 | 2013-04-29 |
| KJ643700 | Bangladesh | A/quail/Bangladesh/19462/2013 | 2013-04-29 |
| KJ643667 | Bangladesh | A/chicken/Bangladesh/19495/2013 | 2013-04-29 |
| KJ643680 | Bangladesh | A/environment/Bangladesh/19301/2013 | 2013-03-30 |
| KJ643597 | Bangladesh | A/chicken/Bangladesh/19870/2013 | 2013-05-25 |
| KJ643672 | Bangladesh | A/environment/Bangladesh/21045/2013 | 2013-10-25 |
| KJ643612 | Bangladesh | A/environment/Bangladesh/19336/2013 | 2013-03-30 |
| KJ643808 | Bangladesh | A/chicken/Bangladesh/18857/2013 | 2013-01-28 |
| KM267815 | Bangladesh | A/chicken/Bangladesh/13/VIR5602-5/2013 | 2013 |
| KM267816 | Bangladesh | A/chicken/Bangladesh/13/VIR5602-6/2013 | 2013 |
| KM267814 | Bangladesh | A/chicken/Bangladesh/13VIR5602-4/2013 | 2013 |
| KJ643739 | Bangladesh | A/chicken/Bangladesh/19565/2013 | 2013-04-29 |
| KJ643726 | Bangladesh | A/quail/Bangladesh/21483/2013 | 2013-12-27 |
| KJ643684 | Bangladesh | A/chicken/Bangladesh/21554/2013 | 2013-12-20 |
| KJ643713 | Bangladesh | A/quail/Bangladesh/21247/2013 | 2013-11-24 |
| KJ643776 | Bangladesh | A/duck/Bangladesh/21126/2013 | 2013-10-25 |
| KM267813 | Bangladesh | A/chicken/Bangladesh/13VIR5602-3/2013 | 2013 |
| KJ643771 | Bangladesh | A/chicken/Bangladesh/19145/2013 | 2013-02-14 |
| KJ643731 | Bangladesh | A/chicken/Bangladesh/20205/2013 | 2013-07-08 |
| KY616779 | Bangladesh | A/chicken/Bangladesh/23727/2014 | 2014-10-19 |
| KY616760 | Bangladesh | A/chicken/Bangladesh/23740/2014 | 2014-10-19 |
| KT362004 | Bangladesh | A/chicken/Bangladesh/23426/2014 | 2014-09-21 |
| KT361962 | Bangladesh | A/environment/Bangladesh/23571/2014 | 2014-10-18 |
| KY616781 | Bangladesh | A/environment/Bangladesh/23641/2014 | 2014-10-18 |
| KT361996 | Bangladesh | A/chicken/Bangladesh/23618/2014 | 2014-10-18 |
| KT362038 | Bangladesh | A/chicken/Bangladesh/23527/2014 | 2014-09-27 |
| KT361972 | Bangladesh | A/chicken/Bangladesh/23529/2014 | 2014-09-27 |
| KY616775 | Bangladesh | A/quail/Bangladesh/24007/2014 | 2014-12-29 |
| KY616750 | Bangladesh | A/quail/Bangladesh/24008/2014 | 2014-12-29 |
| KY635480 | Bangladesh | A/chicken/Bangladesh/27871/2015 | 2015-12-23 |
| KY635751 | Bangladesh | A/chicken/Bangladesh/24249/2015 | 2015-01-23 |
| KY635580 | Bangladesh | A/environment/Bangladesh/24969/2015 | 2015-03-22 |
| KY635538 | Bangladesh | A/environment/Bangladesh/24205/2015 | 2015-01-23 |
| KY635820 | Bangladesh | A/environment/Bangladesh/25969/2015 | 2015-08-25 |
| KY635615 | Bangladesh | A/quail/Bangladesh/24225/2015 | 2015-01-23 |
| KY635445 | Bangladesh | A/chicken/Bangladesh/26102/2015 | 2015-08-27 |
| KY635439 | Bangladesh | A/chicken/Bangladesh/26115/2015 | 2015-08-27 |
| KY635461 | Bangladesh | A/chicken/Bangladesh/26120/2015 | 2015-08-27 |
| KY635479 | Bangladesh | A/chicken/Bangladesh/24947/2015 | 2015-03-22 |
| KY635514 | Bangladesh | A/chicken/Bangladesh/25945/2015 | 2015-08-25 |
| KY635737 | Bangladesh | A/chicken/Bangladesh/25946/2015 | 2015-08-25 |
| KY635534 | Bangladesh | A/chicken/Bangladesh/26223/2015 | 2015-09-12 |
| KY635789 | Bangladesh | A/environment/Bangladesh/26032/2015 | 2015-08-25 |
| KY635657 | Bangladesh | A/environment/Bangladesh/26218/2015 | 2015-09-12 |
| KY635498 | Bangladesh | A/quail/Bangladesh/27835/2015 | 2015-12-23 |
| KY635605 | Bangladesh | A/quail/Bangladesh/24922/2015 | 2015-03-22 |
| KY635749 | Bangladesh | A/quail/Bangladesh/25992/2015 | 2015-08-25 |
| KY635685 | Bangladesh | A/quail/Bangladesh/25997/2015 | 2015-08-25 |
| MG042407 | Bangladesh | A/chicken/Bangladesh/30461/2016 | 2016-07-22 |
| MG042122 | Bangladesh | A/chicken/Bangladesh/30861/2016 | 2016-09-27 |
| MG042175 | Bangladesh | A/chicken/Bangladesh/31066/2016 | 2016-10-24 |
| MG599736 | Bangladesh | A/layer_chicken/Bangladesh/VP05/2016 | 2016-02 |
| KY635684 | Bangladesh | A/chicken/Bangladesh/28182/2016 | 2016-02-05 |
| MG042288 | Bangladesh | A/chicken/Bangladesh/31624/2016 | 2016-12-28 |
| MG042387 | Bangladesh | A/chicken/Bangladesh/29588/2016 | 2016-03-28 |
| MG599733 | Bangladesh | A/layer_chicken/Bangladesh/VP02/2016 | 2016-02 |
| MG599735 | Bangladesh | A/layer_chicken/Bangladesh/VP04/2016 | 2016-02 |
| MG599737 | Bangladesh | A/duck/Bangladesh/VP06/2016 | 2016-02 |
| MG042384 | Bangladesh | A/chicken/Bangladesh/30457/2016 | 2016-07-22 |
| MG042274 | Bangladesh | A/chicken/Bangladesh/30030/2016 | 2016-05-26 |
| MG042217 | Bangladesh | A/quail/Bangladesh/29564/2016 | 2016-03-28 |
| KY635529 | Bangladesh | A/quail/Bangladesh/28122/2016 | 2016-02-03 |
| MG042323 | Bangladesh | A/quail/Bangladesh/30637/2016 | 2016-08-21 |
| MG042386 | Bangladesh | A/quail/Bangladesh/30835/2016 | 2016-09-27 |
| MG042242 | Bangladesh | A/quail/Bangladesh/31043/2016 | 2016-10-24 |
| MG042129 | Bangladesh | A/quail/Bangladesh/31244/2016 | 2016-11-25 |
| MG042305 | Bangladesh | A/quail/Bangladesh/29997/2016 | 2016-05-26 |
| MG957512 | Bangladesh | A/quail/Bangladesh/32525/2017 | 2017-04-19 |
| MG977512 | Bangladesh | A/environment/Bangladesh/32463/2017 | 2017-03-28 |
| EF154913 | China | A/quail/Shantou/2816/2000 | 2000 |
| EF154908 | China | A/quail/Shantou/782/2000 | 2000 |
| EF154909 | China | A/quail/Shantou/1310/2000 | 2000 |
| EF154912 | China | A/quail/Shantou/2061/2000 | 2000 |
| CY024560 | China | A/Guinea_fowl/Shantou/2076/2001 | 2001 |
| CY023096 | China | A/partridge/Shantou/2875/2001 | 2001 |
| EF154923 | China | A/quail/Shantou/2111/2001 | 2001 |
| EF154924 | China | A/quail/Shantou/2462/2001 | 2001 |
| EF154926 | China | A/quail/Shantou/4641/2001 | 2001 |
| EF154917 | China | A/quail/Shantou/1235/2001 | 2001 |
| EF154921 | China | A/quail/Shantou/1555/2001 | 2001 |
| EF154918 | China | A/quail/Shantou/1242/2001 | 2001 |
| EF154914 | China | A/quail/Shantou/222/2001 | 2001 |
| EF154922 | China | A/quail/Shantou/1912/2001 | 2001 |
| EF154936 | China | A/quail/Shantou/1551/2002 | 2002 |
| EF154930 | China | A/quail/Shantou/69/2002 | 2002 |
| EF154937 | China | A/quail/Shantou/3851/2002 | 2002 |
| EF154938 | China | A/quail/Shantou/4203/2002 | 2002 |
| EF154939 | China | A/quail/Shantou/4700/2002 | 2002 |
| EF154942 | China | A/quail/Shantou/335/2003 | 2003 |
| EF154955 | China | A/quail/Shantou/4044/2003 | 2003 |
| EF154948 | China | A/quail/Shantou/3008/2003 | 2003 |
| EF154952 | China | A/quail/Shantou/3768/2003 | 2003 |
| EF154956 | China | A/quail/Shantou/4850/2003 | 2003 |
| EF154949 | China | A/quail/Shantou/3308/2003 | 2003 |
| EF154959 | China | A/quail/Shantou/1475/2004 | 2004 |
| EF154962 | China | A/quail/Shantou/3060/2004 | 2004 |
| EF154960 | China | A/quail/Shantou/1865/2004 | 2004 |
| EF154961 | China | A/quail/Shantou/1883/2004 | 2004 |
| EF154958 | China | A/quail/Shantou/403/2004 | 2004 |
| CY110925 | Egypt | A/chicken/Egypt/S4454B/2011 | 2011-12 |
| CY110927 | Egypt | A/chicken/Egypt/S4454E/2011 | 2011-12 |
| CY110928 | Egypt | A/chicken/Egypt/S4456B/2011 | 2011-12 |
| KC017474 | Egypt | A/chicken/Egypt/A2-D/2011 | 2011-03-20 |
| JQ906551 | Egypt | A/chicken/Egypt/12186F-9/2012 | 2012-02 |
| KF258187 | Egypt | A/chicken/Egypt/D4905A/2012 | 2012-03-12 |
| KF258174 | Egypt | A/chicken/Egypt/S5018A/2012 | 2012-03-25 |
| KF881483 | Egypt | A/chicken/Egypt/S5018C/2012 | 2012-03-25 |
| KF881341 | Egypt | A/chicken/Egypt/D5490B/2012 | 2012-06-04 |
| KF881475 | Egypt | A/chicken/Egypt/S5440E/2012 | 2012-05-26 |
| KF881732 | Egypt | A/chicken/Egypt/S5442E/2012 | 2012-05-26 |
| KF881574 | Egypt | A/chicken/Egypt/S5442C/2012 | 2012-05-26 |
| KF258177 | Egypt | A/chicken/Egypt/Q4698A/2012 | 2012-02-04 |
| JX912990 | Egypt | A/chicken/Egypt/S5018B/2012 | 2012-03-25 |
| JX912997 | Egypt | A/chicken/Egypt/D4692A/2012 | 2012-02-04 |
| JX912984 | Egypt | A/chicken/Egypt/D4907A/2012 | 2012-03-12 |
| KF881394 | Egypt | A/chicken/Egypt/S5018D/2012 | 2012-03-25 |
| JX912985 | Egypt | A/chicken/Egypt/D4905B/2012 | 2012-03-12 |
| KF881459 | Egypt | A/chicken/Egypt/S7404/2013 | 2013-02-18 |
| KF258184 | Egypt | A/chicken/Egypt/D7100/2013 | 2013-01-16 |
| KF258191 | Egypt | A/chicken/Egypt/S7018B/2013 | 2013-01-02 |
| KF258186 | Egypt | A/chicken/Egypt/S7025C/2013 | 2013-01-02 |
| KF881386 | Egypt | A/chicken/Egypt/S7025E/2013 | 2013-01-02 |
| KF881640 | Egypt | A/chicken/Egypt/S7022D/2013 | 2013-01-02 |
| KF881553 | Egypt | A/chicken/Egypt/D7436C/2013 | 2013-02-27 |
| KF258183 | Egypt | A/chicken/Egypt/D7108E/2013 | 2013-01-16 |
| KF881370 | Egypt | A/chicken/Egypt/D7663C/2013 | 2013-04-09 |
| KX000797 | Egypt | A/chicken/Egypt/D9326C/2013 | 2013-12-28 |
| KX000736 | Egypt | A/chicken/Egypt/S9288A/2013 | 2013-12-22 |
| KF258190 | Egypt | A/chicken/Egypt/D7099/2013 | 2013-01-16 |
| KF881378 | Egypt | A/chicken/Egypt/F7297B/2013 | 2013-02-05 |
| KX000830 | Egypt | A/chicken/Egypt/Q10429E/2014 | 2014-12-13 |
| KP027627 | Egypt | A/chicken/Egypt/SCU9/2014 | 2014-02 |
| KP027619 | Egypt | A/chicken/Egypt/SCU8/2014 | 2014-01 |
| KX000760 | Egypt | A/chicken/Egypt/S10312C/2014 | 2014-11-14 |
| KX000789 | Egypt | A/pigeon/Egypt/S10408B/2014 | 2014-12-09 |
| KX000715 | Egypt | A/pigeon/Egypt/S10409A/2014 | 2014-12-09 |
| KP027635 | Egypt | A/chicken/Egypt/SCU20/2014 | 2014-05 |
| KX000864 | Egypt | A/chicken/Egypt/C9742/2014 | 2014-03-04 |
| KX000817 | Egypt | A/chicken/Egypt/D9570B/2014 | 2014-02-11 |
| KX000754 | Egypt | A/chicken/Egypt/S9668D/2014 | 2014-02-17 |
| KX000871 | Egypt | A/chicken/Egypt/D9817E/2014 | 2014-04-11 |
| KX000751 | Egypt | A/duck/Egypt/C9787/2014 | 2014-04-04 |
| KX000845 | Egypt | A/chicken/Egypt/D9377/2014 | 2014-01-15 |
| KX000735 | Egypt | A/chicken/Egypt/Q9394E/2014 | 2014-01-18 |
| KX000748 | Egypt | A/chicken/Egypt/S9348B/2014 | 2014-01-04 |
| KX000758 | Egypt | A/chicken/Egypt/S9645/2014 | 2014-02-17 |
| KX000823 | Egypt | A/chicken/Egypt/S9652E/2014 | 2014-02-17 |
| KX000718 | Egypt | A/chicken/Egypt/F9516B/2014 | 2014-01-15 |
| KX000860 | Egypt | A/chicken/Egypt/F9883C/2014 | 2014-04-23 |
| KX000713 | Egypt | A/chicken/Egypt/F10285D/2014 | 2014-10-26 |
| KX000857 | Egypt | A/quail/Egypt/D9842/2014 | 2014-04-22 |
| KX000790 | Egypt | A/quail/Egypt/D10093/2014 | 2014-08-19 |
| KX000710 | Egypt | A/quail/Egypt/D10105/2014 | 2014-08-19 |
| KX000865 | Egypt | A/quail/Egypt/D10106/2014 | 2014-08-19 |
| KX000844 | Egypt | A/chicken/Egypt/D10561/2015 | 2015-01-27 |
| KX000822 | Egypt | A/chicken/Egypt/D10564/2015 | 2015-01-27 |
| KX000878 | Egypt | A/chicken/Egypt/D10566B/2015 | 2015-01-27 |
| KX000712 | Egypt | A/chicken/Egypt/F10533A/2015 | 2015-01-19 |
| KX000745 | Egypt | A/chicken/Egypt/F10533D/2015 | 2015-01-19 |
| KX000791 | Egypt | A/chicken/Egypt/F10993B/2015 | 2015-04-28 |
| KX000750 | Egypt | A/chicken/Egypt/S10939E/2015 | 2015-04-20 |
| KX000741 | Egypt | A/chicken/Egypt/D10560/2015 | 2015-01-27 |
| KX000838 | Egypt | A/chicken/Egypt/N10963/2015 | 2015-04-24 |
| KX000743 | Egypt | A/chicken/Egypt/N10975/2015 | 2015-04-24 |
| KX000763 | Egypt | A/chicken/Egypt/F10534C/2015 | 2015-01-19 |
| KX000781 | Egypt | A/chicken/Egypt/N10957A/2015 | 2015-04-24 |
| KX000793 | Egypt | A/chicken/Egypt/D10945E/2015 | 2015-04-20 |
| KX000775 | Egypt | A/chicken/Egypt/Q10660B/2015 | 2015-02-21 |
| KX000794 | Egypt | A/chicken/Egypt/S10489C/2015 | 2015-01-05 |
| KT216664 | Egypt | A/chicken/Egypt/S10490/2015 | 2015-01-05 |
| KX000846 | Egypt | A/chicken/Egypt/A1093D/2015 | 2015-02-09 |
| KX000836 | Egypt | A/chicken/Egypt/D10707B/2015 | 2015-02-26 |
| KX000716 | Egypt | A/chicken/Egypt/D10798D/2015 | 2015-03-21 |
| KX000805 | Egypt | A/chicken/Egypt/D10788/2015 | 2015-03-21 |
| KT216663 | Egypt | A/chicken/Egypt/D10802E/2015 | 2015-03-21 |
| KX000853 | Egypt | A/chicken/Egypt/S10916B/2015 | 2015-04-06 |
| KX000708 | Egypt | A/chicken/Egypt/S10597A/2015 | 2015-02-15 |
| KY910830 | Egypt | A/Egypt/ZU63/2016 | 2016-03-02 |
| KY910836 | Egypt | A/Egypt/ZU80/2016 | 2016-03-02 |
| KY558856 | Egypt | A/chicken/Egypt/F12170E/2016 | 2016-03-27 |
| KY558846 | Egypt | A/chicken/Egypt/F12054D/2016 | 2016-05-11 |
| KY558868 | Egypt | A/chicken/Egypt/F12168D/2016 | 2016-03-22 |
| KY558857 | Egypt | A/chicken/Egypt/S12568C/2016 | 2016-05-08 |
| MF289429 | Egypt (Assiut) | A/chicken/Egypt/152537V/2015 | 2015-07 |
| KU296195 | Egypt (El-Beheira) | A/chicken/Egypt/12128VD/2012 | 2012-01-22 |
| KJ781216 | Egypt (El-Beheira) | A/chicken/Egypt/1373Vd/2013 | 2013-01 |
| KU296208 | Egypt (El-Beheira) | A/chicken/Egypt/15255VD/2015 | 2015-04-16 |
| KF998215 | Egypt (Bani Souwaif) | A/chicken/Egypt/BSU-BS-M33/2012 | 2012-07-10 |
| KF998216 | Egypt (Bani Souwaif) | A/chicken/Egypt/BSU-BS-M35/2012 | 2012-07-16 |
| KR010957 | Egypt (Bani Souwaif) | A/chicken/Egypt/BSU-BS-KB59/2013 | 2013-10-12 |
| KU296203 | Egypt (Bani Souwaif) | A/chicken/Egypt/1433RSF/2014 | 2014-12-16 |
| MG662424 | Egypt (Bani Souwaif) | A/chicken/Egypt/ABD9/2015 | 2015 |
| EPI344529 | Egypt (Bani Souwaif) | A/Duck/Egypt/AR526/2017 | 2017-03 |
| EPI344530 | Egypt (Bani Souwaif) | A/Chicken/Egypt/AR528/2017 | 2017-03 |
| EPI344555 | Egypt (Bani Souwaif) | A/Chicken/Egypt/AR551/2018 | 2018-04 |
| MF289428 | Egypt (Dakahliya) | A/chicken/Egypt/158SG/2015 | 2015-01 |
| KU296206 | Egypt (Dakahliya) | A/chicken/Egypt/1560VG/2015 | 2015-02-23 |
| EPI344552 | Egypt (Dakahliya) | A/Chicken/Egypt/AR540/2018 | 2018-01 |
| JX192599 | Egypt (Damitta) | A/chicken/Egypt/111959VG/2011 | 2011-09-08 |
| KU296196 | Egypt (New Valley) | A/chicken/Egypt/13342V/2013 | 2013-02-28 |
| CY126232 | Egypt (Fayoum) | A/chicken/El_Fayoum/CAI25/2011 | 2011-01-01 |
| JQ906556 | Egypt (Fayoum) | A/chicken/Egypt/124B/2012 | 2012-01 |
| JQ906557 | Egypt (Fayoum) | A/chicken/Egypt/1226B/2012 | 2012-02 |
| KF998211 | Egypt (Fayoum) | A/chicken/Egypt/BSU-FA-K6/2012 | 2012-04-02 |
| KF998214 | Egypt (Fayoum) | A/chicken/Egypt/BSU-FA-K9/2012 | 2012-06-11 |
| KF998213 | Egypt (Fayoum) | A/chicken/Egypt/BSU-FA-M28/2012 | 2012-05-02 |
| KF998210 | Egypt (Fayoum) | A/chicken/Egypt/BSU-FA-K4/2012 | 2012-04-02 |
| KF998208 | Egypt (Fayoum) | A/chicken/Egypt/BSU-FA-K2/2012 | 2012-02-25 |
| KF998209 | Egypt (Fayoum) | A/chicken/Egypt/BSU-FA-K3/2012 | 2012-02-25 |
| JQ906558 | Egypt (Fayoum) | A/chicken/Egypt/1231B/2012 | 2012-02 |
| KR010954 | Egypt (Fayoum) | A/chicken/Egypt/BSU-FA-KB1/2012 | 2012-12-04 |
| EPI344526 | Egypt (Fayoum) | A/Duck/Egypt/AR517/2017 | 2017-02 |
| EPI344561 | Egypt (Fayoum) | A/Chicken/Egypt/AR593/2018 | 2018-05 |
| MG662416 | Egypt (Gharbia) | A/chicken/Egypt/ABD1/2015 | 2015 |
| MG662420 | Egypt (Al Gharbya) | A/chicken/Egypt/ABD5/2015 | 2015 |
| MG662422 | Egypt (Al Gharbya) | A/chicken/Egypt/ABD7/2015 | 2015 |
| MG662423 | Egypt (Al Gharbya) | A/chicken/Egypt/ABD8/2015 | 2015 |
| MG662419 | Egypt (Al Gharbya) | A/chicken/Egypt/ABD4/2015 | 2015 |
| JN828570 | Egypt (Giza) | A/quail/Egypt/113413v/2011 | 2011-05-30 |
| JX192600 | Egypt (Giza) | A/chicken/Egypt/115418V/2011 | 2011-10-08 |
| JX192601 | Egypt (Giza) | A/chicken/Egypt/115512V/2011 | 2011-10-15 |
| JQ906553 | Egypt (Giza) | A/chicken/Egypt/115542V/2011 | 2011-10 |
| JX192602 | Egypt (Giza) | A/chicken/Egypt/115583V/2011 | 2011-10-19 |
| JQ906554 | Egypt (Giza) | A/chicken/Egypt/115617V/2011 | 2011-10 |
| KJ781211 | Egypt (Giza) | A/chicken/Egypt/123243V/2012 | 2012-12 |
| KJ781209 | Egypt (Giza) | A/quail/Egypt/122313V/2012 | 2012-09 |
| KJ781215 | Egypt (Giza) | A/chicken/Egypt/13139V/2013 | 2013-01 |
| KJ781214 | Egypt (Giza) | A/turkey/Egypt/1341V/2013 | 2013-01 |
| KU296198 | Egypt (Giza) | A/turkey/Egypt/1386S/2013 | 2013-05-18 |
| MF289425 | Egypt (Giza) | A/chicken/Egypt/1455V/2014 | 2014-01 |
| KU296199 | Egypt (Giza) | A/chicken/Egypt/1474V/2014 | 2014-01-14 |
| KU296202 | Egypt (Giza) | A/quail/Egypt/141267V/2014 | 2014-06-21 |
| KU296201 | Egypt (Giza) | A/quail/Egypt/14864V/2014 | 2014-05-01 |
| KU296209 | Egypt (Giza) | A/quail/Egypt/152792V/2015 | 2015-08-13 |
| EPI344549 | Egypt (Giza) | A/Chicken/Egypt/AR536/2017 | 2017-11 |
| EPI344550 | Egypt (Giza) | A/Chicken/Egypt/AR537/2017 | 2017-11 |
| EPI344551 | Egypt (Giza) | A/Chicken/Egypt/AR538/2017 | 2017-12 |
| EPI344535 | Egypt (Giza) | A/Chicken/Egypt/AR544/2018 | 2018-01 |
| EPI344553 | Egypt (Giza) | A/Chicken/Egypt/AR541/2018 | 2018-01 |
| EPI344556 | Egypt (Giza) | A/Chicken/Egypt/AR554/2018 | 2018-04 |
| EPI344557 | Egypt (Giza) | A/Chicken/Egypt/AR555/2018 | 2018-04 |
| EPI344560 | Egypt (Giza) | A/Chicken/Egypt/AR590/2018 | 2018-05 |
| MF620130 | Egypt (Ismailia) | A/chicken/Egypt/Elfeil-26/2016 | 2016-11-28 |
| EPI344536 | Egypt (Menia) | A/Chicken/Egypt/AR546/2018 | 2018-03 |
| JQ906552 | Egypt (Monofiya) | A/chicken/Egypt/114915V/2011 | 2011-08 |
| KJ781210 | Egypt (Monofiya) | A/chicken/Egypt/123140V/2012 | 2012-10 |
| JQ906560 | Egypt (Monofiya) | A/chicken/Egypt/1240V/2012 | 2012-01 |
| KU296197 | Egypt (Monofiya) | A/chicken/Egypt/13488V/2013 | 2013-03-21 |
| KJ781213 | Egypt (Monofiya) | A/chicken/Egypt/1339V/2013 | 2013-01 |
| KU296204 | Egypt (Monofiya) | A/chicken/Egypt/142712V/2014 | 2014-12-26 |
| MF289426 | Egypt (Monofiya) | A/chicken/Egypt/141794V/2014 | 2014-08 |
| MG662417 | Egypt (Monofiya) | A/chicken/Egypt/ABD2/2015 | 2015 |
| MG662421 | Egypt (Monofiya) | A/chicken/Egypt/ABD6/2015 | 2015 |
| JQ440373 | Egypt (Kalyobiya) | A/chicken/Egypt/114940v/2011 | 2011-08-27 |
| JQ419502 | Egypt (Kalyobiya) | A/chicken/Egypt/114922v/2011 | 2011-08 |
| JQ906555 | Egypt (Kalyobiya) | A/chicken/Egypt/115636V/2011 | 2011-10 |
| KJ781208 | Egypt (Kalyobiya) | A/chicken/Egypt/1256S/2012 | 2012-05 |
| KU296200 | Egypt (Kalyobiya) | A/chicken/Egypt/14246V/2014 | 2014-02-08 |
| MG662418 | Egypt (Kalyobiya) | A/chicken/Egypt/ABD3/2015 | 2015 |
| MG662425 | Egypt (Kalyobiya) | A/chicken/Egypt/ABD10/2015 | 2015 |
| MF434468 | Egypt (Kalyobiya) | A/chicken/Egypt/ME543V/2016 | 2016-02-15 |
| EPI344559 | Egypt (Kalyobiya) | A/Chicken/Egypt/AR589/2018 | 2017-12 |
| EPI344554 | Egypt (Kalyobiya) | A/Chicken/Egypt/AR545/2018 | 2018-03 |
| EPI344558 | Egypt (Kalyobiya) | A/Chicken/Egypt/AR562/2018 | 2018-04 |
| KU296205 | Egypt (Qena) | A/chicken/Egypt/155FAOFL/2015 | 2015-01-26 |
| MF289430 | Egypt (Qena) | A/chicken/Egypt/1685SL/2016 | 2016-05 |
| MF289427 | Egypt (Sharqeia) | A/chicken/Egypt/14153VS/2014 | 2014-09 |
| KJ781207 | Egypt (Sohag) | A/chicken/Egypt/1225VL/2012 | 2012-03 |
| KU296207 | Egypt (Sohag) | A/chicken/Egypt/15106VL/2015 | 2015-04-10 |
| KJ781212 | Egypt (Suez) | A/chicken/Egypt/123245V/2012 | 2012-12 |
| AF156378 | Hong Kong | A/Quail/Hong_Kong/G1/97 | 1997 |
| KY785891 | Hong Kong | A/quail/Hong_Kong/PA-G1/1997 | 1997 |
| KY785893 | Hong Kong | A/quail/Hong_Kong/PA-G1/1997 | 1997 |
| KY785894 | Hong Kong | A/quail/Hong_Kong/PA-G1/1997 | 1997 |
| KY785895 | Hong Kong | A/quail/Hong_Kong/PA-G1/1997 | 1997 |
| KY785892 | Hong Kong | A/quail/Hong_Kong/PA-G1/1997 | 1997 |
| KY785896 | Hong Kong | A/quail/Hong_Kong/G1/1997 | 1997 |
| AJ404626 | Hong Kong | A/Hong_Kong/1073/99 | 1999 |
| AJ404627 | Hong Kong | A/Hong_Kong/1074/1999 | 1999 |
| AY206676 | Hong Kong | A/guinea_fowl/Hong_Kong/WF10/99 | 1999 |
| GU053179 | Hong Kong | A/Hong_Kong/1074/1999 | 1999 |
| CY068643 | India | A/chicken/Chandigarh/2048/2003 | 2003-04-14 |
| JX273542 | India | A/chicken/India/3/2003 | 2003 |
| EU665420 | India | A/chicken/Haryana/2051/2003 | 2003-04-24 |
| EU665421 | India | A/chicken/Punjab/2063/2003 | 2003-04-25 |
| CY068651 | India | A/chicken/Orissa/India/2317/2004 | 2004-02-23 |
| CY068659 | India | A/chicken/Haryana/2424/2004 | 2004-04-12 |
| CY068667 | India | A/chicken/Uttar_Pradesh/2544/2004 | 2004-05-25 |
| CY068672 | India | A/chicken/Gujarat/3697/2004 | 2004-09-13 |
| EU665423 | India | A/chicken/Gujarat/3724/2004 | 2004-10 |
| EU665422 | India | A/chicken/Uttar_Pradesh/2543/2004 | 2004-03 |
| FJ605501 | India | A/watercoot/Haryana/5844/2005 | 2005-10 |
| HM370049 | India | A/chicken/Uchal/8286/2006 | 2006-02-25 |
| HM370056 | India | A/chicken/Uchal/8293/2006 | 2006-02-25 |
| GU064951 | India | A/chicken/Tripura/105131/2008 | 2008-05-29 |
| KM519965 | India | A/chicken/India/80998/2008 | 2008-01-12 |
| KM519973 | India | A/chicken/India/82565/2008 | 2008-01-24 |
| KM922684 | India | A/chicken/India/82588/2008 | 2008-01-24 |
| JX310065 | India | A/chicken/India/WB-NIV1057169/2010 | 2010-10-29 |
| JX310067 | India | A/chicken/India/WB-NIV1057231/2010 | 2010-10-29 |
| JX310066 | India | A/chicken/India/WB-NIV1057183/2010 | 2010-10-29 |
| CY099345 | India | A/chicken/India/IVRI-0004/2010 | 2010-12-06 |
| CY099361 | India | A/chicken/India/IVRI-0011/2011 | 2011-02-01 |
| KT285349 | India | A/chicken/India/01CL1619/2012 | 2012-01-22 |
| KT285341 | India | A/chicken/India/01CL1826/2012 | 2012-01-22 |
| KT285333 | India | A/chicken/India/09CL1359/2013 | 2013-09-23 |
| GQ497118 | Iran | A/chicken/Iran/661/1998 | 1998-10-26 |
| KF800947 | Iran | A/chicken/Iran/av1221/1998 | 1998-02-02 |
| KU357035 | Iran | A/chicken/Iran/12VIR/9630/1998 | 1998 |
| GQ497117 | Iran | A/chicken/Iran/698/1998 | 1998-11-15 |
| FJ794817 | Iran | A/chicken/Iran/SS1/1998 | 1998 |
| EU477241 | Iran | A/chicken/Iran/TH77/1998 | 1998 |
| JX465626 | Iran | A/chicken/Iran/ZMT-101/1998 | 1998-06 |
| JQ419725 | Iran | A/chicken/Iran/725/1998 | 1998 |
| AF508558 | Iran | A/chicken/Iran/11T/99 | 1999 |
| GQ497120 | Iran | A/chicken/Iran/772/1999 | 1999-08-29 |
| EU477247 | Iran | A/chicken/Iran/TH78/1999 | 1999 |
| GQ497119 | Iran | A/chicken/Iran/705/1999 | 1999-10-08 |
| EU477249 | Iran | A/chicken/Iran/TH80/2000 | 2000 |
| GQ497121 | Iran | A/chicken/Iran/565/2000 | 2000-09-14 |
| GQ497122 | Iran | A/chicken/Iran/584/2000 | 2000-07-19 |
| EU477248 | Iran | A/chicken/Iran/TH79/2000 | 2000 |
| AJ781825 | Iran | A/chicken/Iran/AG541/2000 | 2000 |
| GQ497123 | Iran | A/chicken/Iran/233/2001 | 2001-04-09 |
| GQ497124 | Iran | A/chicken/Iran/450/2001 | 2001-07-22 |
| GQ497126 | Iran | A/chicken/Iran/466/2002 | 2002-12-05 |
| GQ497125 | Iran | A/chicken/Iran/284/2002 | 2002-08-13 |
| EU477246 | Iran | A/chicken/Iran/TH81/2002 | 2002 |
| EF063726 | Iran | A/chicken/Iran/B263/2003 | 2003 |
| FN600115 | Iran | A/garganey/Iran/K8/2003 | 2003 |
| GQ497127 | Iran | A/chicken/Iran/92/2003 | 2003-07-17 |
| EF063727 | Iran | A/chicken/Iran/B314/2003 | 2003 |
| EF063725 | Iran | A/chicken/Iran/L252/2003 | 2003 |
| GQ497128 | Iran | A/chicken/Iran/320/2003 | 2003-04-14 |
| DQ922904 | Iran | A/chicken/Iran/L248/2003 | 2003 |
| GQ497130 | Iran | A/chicken/Iran/152/2004 | 2004-11-08 |
| EF063728 | Iran | A/chicken/Iran/B308A/2004 | 2004 |
| EF063730 | Iran | A/chicken/Iran/B322/2004 | 2004 |
| GQ497129 | Iran | A/chicken/Iran/133/2004 | 2004-07-08 |
| EF063731 | Iran | A/chicken/Iran/B76/2004 | 2004 |
| EF063729 | Iran | A/chicken/Iran/B308B/2004 | 2004 |
| GQ497132 | Iran | A/chicken/Iran/119/2005 | 2005-12-21 |
| GQ497131 | Iran | A/chicken/Iran/86/2005 | 2005-11-08 |
| EF063733 | Iran | A/chicken/Iran/B102/2005 | 2005 |
| EF063734 | Iran | A/chicken/Iran/B11A/2005 | 2005 |
| EF063732 | Iran | A/chicken/Iran/B99/2005 | 2005 |
| EF063735 | Iran | A/chicken/Iran/B326/2005 | 2005 |
| GQ497134 | Iran | A/chicken/Iran/68/2006 | 2006-06-09 |
| GQ497133 | Iran | A/chicken/Iran/20/2006 | 2006-11-15 |
| GQ497135 | Iran | A/chicken/Iran/SH1/2007 | 2007-09-01 |
| GU071980 | Iran | A/chicken/Iran/THLBM864/2007 | 2007-11 |
| GQ497136 | Iran | A/chicken/Iran/SH2/2007 | 2007-11-22 |
| EU477245 | Iran | A/chicken/Iran/TH186/2007 | 2007 |
| EU477243 | Iran | A/chicken/Iran/TH286/2007 | 2007 |
| EU477244 | Iran | A/chicken/Iran/TH386/2007 | 2007 |
| EU477539 | Iran | A/chicken/Iran/TH486-masoumi/2007 | 2007 |
| EU477242 | Iran | A/chicken/Iran/TH85/2007 | 2007 |
| GU071982 | Iran | A/chicken/Iran/THLBM866/2007 | 2007-11 |
| GU071979 | Iran | A/chicken/Iran/THLBM863/2007 | 2007-10 |
| GU071983 | Iran | A/chicken/Iran/THLBM867/2007 | 2007-11 |
| GU071984 | Iran | A/chicken/Iran/THLBM868/2007 | 2007-12 |
| GU071981 | Iran | A/chicken/Iran/THLBM865/2007 | 2007-11 |
| GU071978 | Iran | A/chicken/Iran/THLBM862/2007 | 2007-10 |
| FJ794818 | Iran | A/chicken/Iran/SS2/2008 | 2008-12 |
| JX456177 | Iran | A/chicken/Iran/SS3/2008 | 2008-02-19 |
| GU071964 | Iran | A/chicken/Iran/RZ28/2008 | 2008-07-27 |
| GU071965 | Iran | A/chicken/Iran/RZ36/2008 | 2008-09-29 |
| GU071966 | Iran | A/chicken/Iran/RZ37/2008 | 2008-10-12 |
| GU071967 | Iran | A/chicken/Iran/RZ42/2008 | 2008-11-11 |
| GU071968 | Iran | A/chicken/Iran/RZ53/2008 | 2008-12-22 |
| JX456179 | Iran | A/chicken/Iran/SS5/2009 | 2009-02-03 |
| JX456178 | Iran | A/chicken/Iran/SS4/2009 | 2009-01-27 |
| JQ419719 | Iran | A/chicken/Iran/106/2009 | 2009-10 |
| JQ419720 | Iran | A/chicken/Iran/113/2009 | 2009 |
| JQ419721 | Iran | A/chicken/Iran/114/2009 | 2009-11 |
| JQ419718 | Iran | A/chicken/Iran/95/2009 | 2009-07 |
| JQ419722 | Iran | A/chicken/Iran/119/2009 | 2009-11 |
| JQ419724 | Iran | A/chicken/Iran/192/2009 | 2009-11 |
| JQ419723 | Iran | A/chicken/Iran/121/2009 | 2009-11 |
| JN646748 | Iran | A/chicken/Iran/EBGV-88/2010 | 2010-02 |
| JX456180 | Iran | A/chicken/Iran/SS6/2010 | 2010-12-25 |
| KF800939 | Iran | A/chicken/Iran/187/2011 | 2011-03-04 |
| JQ970436 | Iran | A/chicken/Iran/N101/2011 | 2011-01-06 |
| JQ970437 | Iran | A/chicken/Iran/N102/2011 | 2011-02-09 |
| JX456182 | Iran | A/chicken/Iran/SS8/2011 | 2011-02-06 |
| JX456181 | Iran | A/chicken/Iran/SS7/2011 | 2011-11-17 |
| KF800938 | Iran | A/chicken/Iran/186/2011 | 2011-02-03 |
| KF800940 | Iran | A/chicken/Iran/189/2011 | 2011-06-05 |
| JX294920 | Iran | A/chicken/Iran/AGH-B1/2012 | 2012-02-12 |
| JX294923 | Iran | A/chicken/Iran/AGH-B4/2012 | 2012-03-12 |
| JX294922 | Iran | A/chicken/Iran/AGH-B3/2012 | 2012-03-10 |
| JX294921 | Iran | A/chicken/Iran/AGH-B2/2012 | 2012-02-20 |
| KJ696533 | Iran | A/chicken/Iran/Markazi/2013 | 2013-07-02 |
| KJ696534 | Iran | A/chicken/Iran/Markazi/2014 | 2014-01-27 |
| MF114292 | Iran | A/chicken/Iran/D1/2016 | 2016 |
| MF114293 | Iran | A/chicken/Iran/D7/2016 | 2016 |
| JX273543 | Iraq | A/chicken/Iraq/EKI_14/2008 | 2008 |
| EF492221 | Israel | A/chicken/Israel/90658/2000 | 2000 |
| AY738451 | Israel | A/turkey/Neve_Ilan/90710/2000 | 2000-5-30 |
| AY738454 | Israel | A/chicken/Tel_Adashim/786/2001 | 2001-12-07 |
| EF492233 | Israel | A/turkey/Israel/810/2001 | 2001 |
| AY738452 | Israel | A/turkey/Givat_Haim/965/2002 | 2002-3-17 |
| EF492229 | Israel | A/turkey/Israel/619/2002 | 2002 |
| EF492237 | Israel | A/turkey/Israel/1013/2002 | 2002 |
| AY738456 | Israel | A/ostrich/Eshkol/1436/2003 | 2003-10-30 |
| EF492238 | Israel | A/turkey/Israel/1209/2003 | 2003 |
| EF492239 | Israel | A/chicken/Israel/1376/2003 | 2003 |
| AY738455 | Israel | A/chicken/Talmei_Elazar/1304/03 | 2003-5-05 |
| EF492240 | Israel | A/chicken/Israel/1475/2003 | 2003 |
| JX273544 | Israel | A/chicken/Israel/421201/2004 | 2004 |
| AY738453 | Israel | A/turkey/Beit_HaLevi/1562/04 | 2004-12-28 |
| EF492242 | Israel | A/chicken/Israel/1808/2004 | 2004 |
| EF501983 | Israel | A/chicken/Israel/1953/2004 | 2004 |
| EF492241 | Israel | A/turkey/Israel/1567/2004 | 2004 |
| EF492243 | Israel | A/chicken/Israel/1966/2004 | 2004 |
| EF492228 | Israel | A/avian/Israel/584/2005 | 2005 |
| EF492227 | Israel | A/chicken/Israel/554/2005 | 2005 |
| EF492223 | Israel | A/turkey/Israel/89/2005 | 2005 |
| EF492222 | Israel | A/chicken/Israel/29/2005 | 2005 |
| EF492225 | Israel | A/chicken/Israel/282/2005 | 2005 |
| EF492226 | Israel | A/turkey/Israel/425/2005 | 2005 |
| EF492230 | Israel | A/turkey/Israel/747/2005 | 2005 |
| EF492235 | Israel | A/turkey/Israel/884/2005 | 2005 |
| EF492232 | Israel | A/chicken/Israel/793/2005 | 2005 |
| EF492234 | Israel | A/chicken/Israel/853/2005 | 2005 |
| EF492236 | Israel | A/chicken/Israel/909/2005 | 2005 |
| EF492224 | Israel | A/chicken/Israel/178/2006 | 2006 |
| FJ464728 | Israel | A/chicken/Israel/1525/2006 | 2006-12-10 |
| FJ464729 | Israel | A/chicken/Israel/1548/2006 | 2006-12-13 |
| FJ464714 | Israel | A/chicken/Israel/1638/2006 | 2006-12-26 |
| FJ464730 | Israel | A/turkey/Israel/1608/2006 | 2006 |
| FJ464715 | Israel | A/chicken/Israel/114/2007 | 2007-01-11 |
| GQ120553 | Israel | A/chicken/Israel/375/2007 | 2007-03-06 |
| FJ464727 | Israel | A/chicken/Israel/1033/2007 | 2007-12-21 |
| FJ464725 | Israel | A/turkey/Israel/900/2007 | 2007 |
| GQ120561 | Israel | A/chicken/Israel/1040/2007 | 2007-12-23 |
| GQ120557 | Israel | A/chicken/Israel/869/2007 | 2007-11-04 |
| GQ120559 | Israel | A/chicken/Israel/951/2007 | 2007-12-02 |
| FJ464726 | Israel | A/chicken/Israel/933/2007 | 2007-11-26 |
| GQ120560 | Israel | A/chicken/Israel/953/2007 | 2007-11-29 |
| FJ464720 | Israel | A/chicken/Israel/386/2007 | 2007-03-07 |
| FJ464722 | Israel | A/chicken/Israel/449/2007 | 2007-04-04 |
| FJ464721 | Israel | A/chicken/Israel/402/2007 | 2007-03-14 |
| FJ464716 | Israel | A/chicken/Israel/215/2007 | 2007-02-01 |
| FJ464724 | Israel | A/chicken/Israel/728/2007 | 2007-07-26 |
| FJ464718 | Israel | A/avian/Israel/313/2008 | 2008 |
| FJ464719 | Israel | A/avian/Israel/314/2008 | 2008 |
| FJ464717 | Israel | A/chicken/Israel/292/2008 | 2008-01-25 |
| FJ464723 | Israel | A/chicken/Israel/524/2008 | 2008-03-09 |
| GQ120552 | Israel | A/chicken/Israel/330/2008 | 2008-06-29 |
| GQ120556 | Israel | A/chicken/Israel/702/2008 | 2008-04-21 |
| GQ120554 | Israel | A/turkey/Israel/689/2008 | 2008-04-15 |
| GQ120551 | Israel | A/chicken/Israel/310/2008 | 2008-06-27 |
| GQ120555 | Israel | A/chicken/Israel/694/2008 | 2008-04-18 |
| GQ120558 | Israel | A/chicken/Israel/883/2008 | 2008-06-13 |
| JQ347951 | Israel | A/chicken/Israel/760/2008 | 2008-05 |
| JQ347954 | Israel | A/chicken/Israel/884/2008 | 2008-06 |
| JQ973661 | Israel | A/chicken/Israel/1205/2008 | 2008-12 |
| JQ347956 | Israel | A/chicken/Israel/1195/2008 | 2008-11 |
| JQ347955 | Israel | A/chicken/Israel/1018/2008 | 2008-10 |
| JQ347958 | Israel | A/chicken/Israel/1256/2008 | 2008-12 |
| GQ120549 | Israel | A/chicken/Israel/182/2008 | 2008-06-10 |
| GQ120548 | Israel | A/chicken/Israel/54/2008 | 2008-06-01 |
| JQ347959 | Israel | A/turkey/Israel/1257/2008 | 2008-12 |
| JQ347957 | Israel | A/turkey/Israel/1223/2008 | 2008-12 |
| JQ254937 | Israel | A/chicken/Israel/32/2009 | 2009-06 |
| GQ120550 | Israel | A/chicken/Israel/184/2009 | 2009-02-15 |
| JQ347946 | Israel | A/chicken/Israel/663/2009 | 2009-11 |
| JQ347945 | Israel | A/turkey/Israel/117/2009 | 2009-01 |
| JQ254940 | Israel | A/turkey/Israel/311/2009 | 2009-03 |
| JQ347953 | Israel | A/chicken/Israel/794/2009 | 2009-12 |
| JQ347943 | Israel | A/chicken/Israel/9/2009 | 2009-01 |
| JQ347947 | Israel | A/chicken/Israel/712/2009 | 2009-11 |
| JQ347952 | Israel | A/turkey/Israel/775/2009 | 2009-12 |
| JQ347949 | Israel | A/chicken/Israel/742/2009 | 2009-11 |
| JQ347950 | Israel | A/turkey/Israel/746/2009 | 2009-11 |
| JQ347948 | Israel | A/chicken/Israel/724/2009 | 2009-11 |
| JQ254945 | Israel | A/chicken/Israel/1167/2010 | 2010-11 |
| JQ254952 | Israel | A/chicken/Israel/1293/2010 | 2010-12 |
| JQ254948 | Israel | A/chicken/Israel/1184/2010 | 2010-11 |
| JQ254942 | Israel | A/chicken/Israel/1067/2010 | 2010-10 |
| JQ254954 | Israel | A/chicken/Israel/1302/2010 | 2010-12 |
| JQ973657 | Israel | A/chicken/Israel/1089/2011 | 2011-12 |
| JQ973659 | Israel | A/chicken/Israel/1163/2011 | 2011-12 |
| JQ973658 | Israel | A/chicken/Israel/1115/2011 | 2011-12 |
| JQ973660 | Israel | A/chicken/Israel/1164/2011 | 2011-12 |
| JQ973652 | Israel | A/chicken/Israel/11/2012 | 2012-01 |
| JQ973653 | Israel | A/chicken/Israel/46/2012 | 2012-01 |
| JQ973651 | Israel | A/chicken/Israel/3/2012 | 2012-01 |
| JQ973654 | Israel | A/chicken/Israel/50/2012 | 2012-01 |
| JQ973656 | Israel | A/chicken/Israel/58/2012 | 2012-01 |
| KF918708 | Israel | A/chicken/Israel/502/2013 | 2013-04 |
| KJ557348 | Israel | A/chicken/Israel/509/2013 | 2013-04-15 |
| KJ739320 | Israel | A/chicken/Israel/626/2013 | 2013-05-07 |
| KJ739323 | Israel | A/chicken/Israel/636/2013 | 2013-05-13 |
| KJ557350 | Israel | A/chicken/Israel/642/2013 | 2013-05-07 |
| KJ739315 | Israel | A/chicken/Israel/443/2013 | 2013-03-07 |
| KJ739317 | Israel | A/chicken/Israel/499/2013 | 2013-04-04 |
| KJ739314 | Israel | A/chicken/Israel/239/2013 | 2013-01-27 |
| KJ739316 | Israel | A/chicken/Israel/465/2013 | 2013-03-21 |
| KJ739318 | Israel | A/chicken/Israel/623/2013 | 2013-05-05 |
| KF918709 | Israel | A/chicken/Israel/74/2013 | 2013-01 |
| KJ557349 | Israel | A/chicken/Israel/510/2013 | 2013-04-15 |
| KJ739319 | Israel | A/chicken/Israel/625/2013 | 2013-05-07 |
| KJ739322 | Israel | A/chicken/Israel/635/2013 | 2013-05-13 |
| KJ739324 | Israel | A/chicken/Israel/802/2013 | 2013-07-01 |
| KJ557351 | Israel | A/turkey/Israel/643/2013 | 2013-05-16 |
| KJ739321 | Israel | A/chicken/Israel/628/2013 | 2013-05-10 |
| KJ831046 | Israel | A/chicken/Israel/45/2014 | 2014-01-12 |
| KJ831050 | Israel | A/chicken/Israel/203/2014 | 2014-03-06 |
| KJ831052 | Israel | A/turkey/Israel/182/2014 | 2014-03-02 |
| KJ831051 | Israel | A/chicken/Israel/199/2014 | 2014-03-04 |
| KJ831053 | Israel | A/chicken/Israel/130/2014 | 2014-02-13 |
| KJ831055 | Israel | A/turkey/Israel/69/2014 | 2014-01-19 |
| KJ831059 | Israel | A/chicken/Israel/1/2014 | 2014-01-01 |
| KJ831058 | Israel | A/chicken/Israel/14/2014 | 2014-01-06 |
| KJ831057 | Israel | A/chicken/Israel/46/2014 | 2014-01-12 |
| KJ831056 | Israel | A/chicken/Israel/68/2014 | 2014-01-21 |
| KJ831054 | Israel | A/chicken/Israel/82/2014 | 2014-01-28 |
| KJ831049 | Israel | A/chicken/Israel/246/2014 | 2014-03-17 |
| KJ831048 | Israel | A/chicken/Israel/254/2014 | 2014-03-21 |
| KJ831047 | Israel | A/chicken/Israel/258/2014 | 2014-03-20 |
| KX783419 | Israel | A/chicken/Israel/451/2016 | 2016-06-06 |
| KX783414 | Israel | A/chicken/Israel/130/2016 | 2016-02-10 |
| KX783417 | Israel | A/chicken/Israel/214/2016 | 2016-04-11 |
| KX783415 | Israel | A/chicken/Israel/54/2016 | 2016-01-31 |
| KX783416 | Israel | A/turkey/Israel/241/2016 | 2016-04-21 |
| KX783418 | Israel | A/chicken/Israel/461/2016 | 2016-06-16 |
| KX783420 | Israel | A/chicken/Israel/450/2016 | 2016-06-10 |
| MF673359 | Jordan | A/chicken/Jordan/14/2003 | 2003-12-11 |
| MF673343 | Jordan | A/chicken/Jordan/12/2003 | 2003-11-18 |
| MF673319 | Jordan | A/chicken/Jordan/13/2003 | 2003-12-11 |
| MF673375 | Jordan | A/chicken/Jordan/47/2003 | 2003-03-08 |
| MF673367 | Jordan | A/chicken/Jordan/10/2003 | 2003-11-18 |
| MF673383 | Jordan | A/chicken/Jordan/11/2003 | 2003-11-18 |
| MF673351 | Jordan | A/chicken/Jordan/56/2003 | 2003-03-05 |
| JX273537 | Jordan | A/avian/Jordan/7-Y1/2004 | 2004 |
| MF673327 | Jordan | A/chicken/Jordan/45/2004 | 2004-04-26 |
| MF673311 | Jordan | A/chicken/Jordan/70/2004 | 2004-10-28 |
| MF673303 | Jordan | A/chicken/Jordan/55/2004 | 2004-04-26 |
| MF673335 | Jordan | A/chicken/Jordan/88/2005 | 2005-02-16 |
| JX273546 | Lebanon | A/chicken/Lebanon/1080/2004 | 2004 |
| CY093088 | Lebanon | A/quail/Lebanon/272/2010 | 2010-08 |
| CY093096 | Lebanon | A/quail/Lebanon/273/2010 | 2010-08 |
| JX273538 | Libya | A/avian/Libya/RV35D/2006 | 2006 |
| KM244119 | Libya | A/chicken/Libya/13VIR7225-2/2013 | 2013 |
| KM244121 | Libya | A/chicken/Libya/13VIR7225-5/2013 | 2013 |
| MG831955 | Morocco | A/broiler_chicken/Casablanca/16VIR9564-1/2016 | 2016-02 |
| LT598512 | Morocco | A/chicken/Morocco/SF1/2016 | 2016 |
| LT598514 | Morocco | A/chicken/Morocco/SF3/2016 | 2016 |
| LT598513 | Morocco | A/chicken/Morocco/SF2/2016 | 2016 |
| LT598516 | Morocco | A/chicken/Morocco/SF5/2016 | 2016 |
| LT598515 | Morocco | A/chicken/Morocco/SF4/2016 | 2016 |
| AJ781823 | Pakistan | A/chicken/Pakistan/AG519/98 | 1999 |
| KF188299 | Pakistan | A/chicken/Pakistan/2/1999 | 1999 |
| AF508556 | Pakistan | A/chicken/Pakistan/5/99 | 1999 |
| AJ291392 | Pakistan | A/chicken/Pakistan/2/1999 | 1999 |
| AF508555 | Pakistan | A/chicken/Pakistan/4/99 | 1999 |
| JX273553 | Pakistan | A/chicken/G-Karachi/2003 | 2003 |
| KX759091 | Pakistan | A/chicken/Abbottabad/NARC-04N-233/2004 | 2004-06-02 |
| KF188399 | Pakistan | A/chicken/Faisalabad/NARC-805/2004 | 2004 |
| KX759089 | Pakistan | A/chicken/Sihala/NARC-04N-087/2004 | 2004-04-22 |
| KF188347 | Pakistan | A/chicken/Pakistan/NARC-1624/2005 | 2005 |
| KX759095 | Pakistan | A/crow/Tarbela/NARC-05N-696/2005 | 2005-11-12 |
| CY038410 | Pakistan | A/chicken/Pakistan/UDL-01/2005 | 2005-05-18 |
| KF188403 | Pakistan | A/chicken/Pakistan/NARC-1559/2005 | 2005 |
| KX759097 | Pakistan | A/coot/Sanghar/NARC-05N-753/2005 | 2005-11-21 |
| KF188359 | Pakistan | A/chicken/Pakistan/NARC-1541/2005 | 2005 |
| KF188325 | Pakistan | A/chicken/Pakistan/NARC-1617/2005 | 2005 |
| CY038418 | Pakistan | A/chicken/Pakistan/UDL-02/2005 | 2005-12-31 |
| CY038442 | Pakistan | A/chicken/Pakistan/UDL-03/2005 | 2005-12-21 |
| KX759093 | Pakistan | A/egret/Tarbela/NARC-05N-694/2005 | 2005-11-12 |
| JX273554 | Pakistan | A/chicken/Pakistan/NARC-2434/2006 | 2006 |
| JN540058 | Pakistan | A/chicken/Sawabi/NARC-2434/2006 | 2006-02-23 |
| CY038426 | Pakistan | A/chicken/Pakistan/UDL-01/2006 | 2006-05-02 |
| CY038402 | Pakistan | A/chicken/Pakistan/UDL-04/2006 | 2006-06-19 |
| CY038434 | Pakistan | A/chicken/Pakistan/UDL-02/2006 | 2006-12-29 |
| CY038482 | Pakistan | A/chicken/Pakistan/UDL-04/2007 | 2007-03-23 |
| CY038450 | Pakistan | A/chicken/Pakistan/UDL-03/2007 | 2007-12-17 |
| CY038394 | Pakistan | A/chicken/Pakistan/UDL-01/2007 | 2007-06-07 |
| JN540066 | Pakistan | A/chicken/Sihala/NARC-12103/2008 | 2008-02-23 |
| CY038466 | Pakistan | A/chicken/Pakistan/UDL-02/2008 | 2008-02-22 |
| CY038474 | Pakistan | A/chicken/Pakistan/UDL-03/2008 | 2008-03-04 |
| JN540074 | Pakistan | A/chicken/Attock/NARC-14994/2009 | 2009-12-03 |
| KF975463 | Pakistan | A/chicken/Pakistan/283-11/2010 | 2010-01-23 |
| KF975464 | Pakistan | A/chicken/Pakistan/284-48/2010 | 2010-01-25 |
| KF975466 | Pakistan | A/chicken/Pakistan/286-65/2010 | 2010-01-26 |
| KF975467 | Pakistan | A/chicken/Pakistan/287-98/2010 | 2010-01-27 |
| KF975468 | Pakistan | A/chicken/Pakistan/288-102/2010 | 2010-01-27 |
| KF975472 | Pakistan | A/chicken/Pakistan/291-266/2010 | 2010-02-14 |
| KF975470 | Pakistan | A/chicken/Pakistan/294-199/2010 | 2010-02-11 |
| KF975465 | Pakistan | A/chicken/Pakistan/285-63/2010 | 2010-01-26 |
| JQ905259 | Pakistan | A/chicken/Pakistan/MM81/2010 | 2010-04-10 |
| KF975469 | Pakistan | A/chicken/Pakistan/289-186/2010 | 2010-02-11 |
| KF975471 | Pakistan | A/chicken/Pakistan/290-230/2010 | 2010-02-13 |
| KU042894 | Pakistan | A/chicken/Pakistan/26A/2012 | 2012-01-01 |
| KU042910 | Pakistan | A/chicken/Pakistan/10A/2015 | 2015-05-11 |
| MG720814 | Pakistan | A/chicken/Pakistan/660BYP/2015 | 2015-05-19 |
| CY081264 | Saudi Arabia | A/chicken/Saudi_Arabia/CP7/1998 | 1998 |
| AF508559 | Saudi Arabia | A/chicken/Saudi_Arabia/532/99 | 1999 |
| GU050554 | Saudi Arabia | A/chicken/Saudi_Arabia/EPD-22-01/2002 | 2002 |
| JX273556 | Saudi Arabia | A/chicken/Saudi_Arabia/582/2005 | 2005 |
| GU050279 | Saudi Arabia | A/avian/Saudi_Arabia/910134/2006 | 2006 |
| GU050295 | Saudi Arabia | A/avian/Saudi_Arabia/910136/2006 | 2006 |
| JX273555 | Saudi Arabia | A/chicken/Saudi_Arabia/2BL09/2006 | 2006 |
| JX273560 | Saudi Arabia | A/chicken/Saudi_Arabia/F-36365/2006 | 2006 |
| JX273559 | Saudi Arabia | A/chicken/Saudi_Arabia/E-36364/2006 | 2006 |
| GU050287 | Saudi Arabia | A/avian/Saudi_Arabia/910135/2006 | 2006 |
| JX273557 | Saudi Arabia | A/chicken/Saudi_Arabia/C-36362/2010 | 2010 |
| JX273558 | Saudi Arabia | A/chicken/Saudi_Arabia/D-36363/2010 | 2010 |
| JF323006 | Tunisia | A/chicken/Tunisia/12/2010 | 2010-01 |
| JF323007 | Tunisia | A/migratory_bird/Tunisia/51/2010 | 2010-01 |
| JQ952588 | Tunisia | A/chicken/Tunisia/2019/2010 | 2010-03 |
| JQ952589 | Tunisia | A/turkey/Tunisia/2068/2010 | 2010-12 |
| JQ952590 | Tunisia | A/chicken/Tunisia/345/2011 | 2011-01 |
| JQ952591 | Tunisia | A/chicken/Tunisia/848/2011 | 2011-01 |
| KU662356 | Tunisia | A/chicken/Tunisia/56/2014 | 2014-02-15 |
| AJ781824 | United Arab Emirates | A/chicken/United_Arab_Emirates/AG537/99 | 1999 |
| EF063510 | United Arab Emirates | A/quail/Dubai/301/2000 | 2000 |
| EF063511 | United Arab Emirates | A/quail/Dubai/302/2000 | 2000 |
| EF063512 | United Arab Emirates | A/quail/Dubai/303/2000 | 2000 |
| EF063513 | United Arab Emirates | A/chicken/Dubai/338/2001 | 2001 |
| KF188352 | United Arab Emirates | A/chicken/Dubai/339/2001 | 2001 |
| EF063514 | United Arab Emirates | A/chicken/Dubai/339/2001 | 2001 |
| EF063515 | United Arab Emirates | A/chicken/Dubai/383/2002 | 2002 |
| CY076723 | United Arab Emirates | A/chicken/Emirates/R66/2002 | 2002 |
| EF063516 | United Arab Emirates | A/chicken/Dubai/463/2003 | 2003 |
| KF188254 | United Arab Emirates | A/quail/United_Arab_Emirates/1136/2005 | 2005 |
| KF188337 | United Arab Emirates | A/stone_curlew/United_Arab_Emirates/1147/2005 | 2005 |
| KF188371 | United Arab Emirates | A/stone_curlew/United_Arab_Emirates/1127.3/2006 | 2005 |
| KF188311 | United Arab Emirates | A/poultry/United_Arab_Emirates/1831/2005 | 2005 |
| KF188258 | United Arab Emirates | A/white_bellied_bustard/United_Arab_Emirates/1019/2005 | 2005 |
| KF188260 | United Arab Emirates | A/white_bellied_bustard/United_Arab_Emirates/997/2005 | 2005 |
| KF188236 | United Arab Emirates | A/white_bellied_bustard/United_Arab_Emirates/1036/2005 | 2005 |
| KF188244 | United Arab Emirates | A/white_bellied_bustard/United_Arab_Emirates/1127.1/2005 | 2005 |
| KF188375 | United Arab Emirates | A/quail/United_Arab_Emirates/1819/2006 | 2006 |
| KF188329 | United Arab Emirates | A/houbara/United_Arab_Emirates/78/2006 | 2006 |
| KF188238 | United Arab Emirates | A/dove/United_Arab_Emirates/466/2006 | 2006 |
| KF188240 | United Arab Emirates | A/falcon/United_Arab_Emirates/897/2007 | 2005 |
| KC555105 | United Arab Emirates | A/pheasant/United_Arab_Emirates/D1307.B/2011 | 2011-06-15 |
| KC555089 | United Arab Emirates | A/pheasant/United_Arab_Emirates/D1521/2011 | 2011-07-24 |
| KC555081 | United Arab Emirates | A/white/bellied_bustard/United_Arab_Emirates/D1520/2011 | 2011-07-24 |
| KC555097 | United Arab Emirates | A/quail/United_Arab_Emirates/D1556/2011 | 2011-08-01 |
| KX351198 | United Arab Emirates | A/chicken/Dubai/D2506.A/2015 | 2015-11-27 |

*Viruses in bold were generated by the National Laboratory for Veterinary Quality Control on Poultry Production, Animal Health Research Institute
